# Oncolytic viruses: a promising therapy for malignant pleural effusion and solid tumors

**DOI:** 10.3389/fimmu.2025.1570698

**Published:** 2025-04-25

**Authors:** Xinya Wang, Qin Zhou, Xuyan Zhang, Han Hu, Binlei Liu, Yang Wang

**Affiliations:** ^1^ National “111” Center for Cellular Regulation and Molecular Pharmaceutics, Key Laboratory of Fermentation Engineering (Ministry of Education), Cooperative Innovation Center of Industrial Fermentation (Ministry of Education & Hubei Province), School of Life and Health Sciences, Hubei University of Technology, Wuhan, China; ^2^ Wuhan Binhui Biopharmaceutical Co., Ltd., Wuhan, China

**Keywords:** oncolytic virus, malignant pleural effusion, tumor microenvironment, solid tumor, intracavitary administration

## Abstract

Oncolytic viruses (OVs) are natural or recombinant viruses that can directly lyse tumor cells without damaging normal cells. They enhance anti-tumor immunity by releasing antigens and activating inflammatory responses within the tumor microenvironment (TME). This offers a new therapeutic approach for MPE and solid tumors. This review discusses the progress of OVs administered via intrapleural and intratumoral routes, emphasizing their potential in MPE treatment and the challenges posed by the complex intrapleural environment, which affects the direct interaction between OVs, tumor cells, and immune cells. This review also discusses the regulatory barriers, safety concerns and accessibility of oncolytic virus therapy.

## 1 Introduction

Oncolytic viruses (OVs), either naturally occurring or genetically engineered, are emerging cancer immunotherapies that selectively replicate in tumor cells, leading to tumor cell lysis (1). OVs exert their antitumor effects through several mechanisms, including selective replication in tumor cells (2), induction of immunogenic cell death (3, 4), targeting tumor vasculature (5), and genetic modification to enhance specificity and efficacy (6, 7).

The most common route of administration for oncolytic viruses (OVs) is intratumoral injection (8). The approved OVs, T-VEC and G47Δ, have demonstrated good safety and efficacy following intratumoral administration (9, 10). Additionally, multiple clinical trials have shown that intratumoral injection not only reduces the size of injected tumors but also decreases the size of distant, non-injected tumors, leading to prolonged patient survival (11, 12). These findings indicate that OVs, in addition to directly lysing tumor cells, can also modulate local antitumor immunity within the tumor microenvironment.

Intracavitary administration serves as an intermediate route between intratumoral and intravenous administration. Compared to intratumoral injection, intracavitary administration allows OVs to interact more broadly with tumor and immune cells while being relatively easier to perform. Compared to systemic intravenous administration, intracavitary delivery achieves higher local viral concentrations in the tumor microenvironment while minimizing potential systemic adverse effects (13). Therefore, intracavitary administration is an important delivery method for OVs and has demonstrated promising efficacy in several clinical trials (14–16).

Malignant pleural effusion (MPE), affecting 8-15% of cancer patients, is mainly caused by lung and breast cancers (17–19). It leads to poor quality of life and a median survival of 3-12 months. Current treatments are primarily palliative (20, 21).

Effective treatment of malignant pleural effusion (MPE) requires meeting three critical conditions: killing tumor cells in the effusion (22), activating the antitumor immunity of lymphocytes in the effusion (23), and repairing damaged blood vessels and lymphatic vessels (24, 25). OVs have shown potential in addressing these aspects, making them a promising therapeutic option for MPE.

This review discusses the progress of OVs in monotherapy and combination therapies, focusing on intratumoral and intrapleural administration as well as their application in MPE treatment. Additionally, it explores the regulatory challenges, cost considerations, safety concerns, and accessibility of OV therapies.

## 2 Mechanisms, combination strategies, and delivery methods of oncolytic viruses

Oncolytic viruses (OVs), a novel class of cancer immunotherapy agents, have garnered increasing attention. OVs include both naturally occurring and genetically engineered viruses capable of selectively replicating in tumor cells, leading to tumor cell lysis and immunogenic tumor cell death (1). OVs exert their antitumor effects through several mechanisms: Selective replication in tumor cells: OVs specifically target and replicate within tumor cells (2). Induction of immunogenic cell death: OVs kill tumor cells, releasing tumor antigens that activate dendritic cells, enhance T-cell infiltration, recruit immune-related molecules, and transform “cold” tumors into “hot” tumors, ultimately leading to the eradication of distant, uninfected tumor cells (3, 4). Targeting tumor vasculature: OVs can infect and disrupt the tumor vascular system, causing neutrophil infiltration, vascular collapse, and tumor cell death (5). Genetic modification: OVs can be genetically engineered to delete genes that recognize normal cells and to insert genes that enhance the antitumor response, thereby improving their specificity and efficacy against certain tumor cells (6, 7).

Oncolytic viruses play an important role in directly killing tumor cells and activating the anti-tumor immunity of immune cells (26, 27). Therefore, the combination of oncolytic viruses with immune checkpoint inhibitors, chemotherapy, and radiotherapy can enhance their anti-tumor efficacy (28).

Oncolytic viruses combined with immune checkpoint inhibitors (ICIs): Oncolytic viruses can upregulate the expression of immune checkpoint molecules, such as PD-1/PD-L1, NKG2A/HLA-E, and others, on the surface of immune and tumor cells. This upregulation provides potential therapeutic targets for subsequent combination with immune checkpoint inhibitors (29). However, OVs with ICIs requires careful consideration of the anti-viral immune response induced by T-cell activation. The activation of T cells by ICIs may enhance anti-viral immunity, potentially leading to the premature clearance of OVs, thereby compromising their therapeutic efficacy. The sequence and timing of administration of OVs and ICIs are critical factors for the success of this combination. Proper scheduling can maximize anti-tumor effects while minimizing the risk of OV clearance by activated T cells (30).

Oncolytic viruses combined with Radiotherapy: Both OVs and radiotherapy can induce immunogenic cell death (ICD) in tumor cells, leading to increased release of tumor-associated antigens (TAAs), enhanced antigen presentation by antigen-presenting cells (APCs), and activation of T cells (31). However, it is important to consider the potential immunosuppressive effects induced by radiotherapy. Radiation can alter the tumor microenvironment (TME) and suppress the function of immune cells, which may attenuate the efficacy of OVs.

Oncolytic viruses combined with chemotherapy: chemotherapy indices direct tumor cell death through DNA damage, thereby enhancing the oncolytic effects of OVs. Furthermore, chemotherapy can selectively deplete immunosuppressive cells such as regulatory T cells (Tregs) and myeloid-derived suppressor cells (MDSCs) (32, 33), thereby reversing immune tolerance in the TME and facilitating OV replication and spread. OVs can further induce apoptosis and autophagy in tumor cells, targeting specific signaling pathways (such as NF-κB and PI3K/AKT) involved in chemotherapy resistance, ultimately enhancing chemosensitivity and overcoming tumor resistance (34).

Common delivery methods for OVs include: 1) Intratumoral Injection: This involves directly injecting OVs into the tumor, producing a localized therapeutic effect. This method allows precise control of OV concentration at the target site, reducing off-target effects and related adverse events. Intratumoral injection offers significant advantages in maintaining optimal OV concentrations at the tumor site, often resulting in clearer therapeutic outcomes. Researchers can better correlate *in vitro* and *in vivo* results using this method. However, the technical challenges associated with administration make it more suitable for superficial tumors like melanoma rather than deeper tumors such as glioblastoma. This limitation also hinders repeated administration of OVs. 2) Intravenous Injection: After injection into peripheral veins, OVs travel through the circulatory system to reach tumor lesions in nonspecific organs or systems. Compared to intratumoral injection, intravenous delivery is simpler and can overcome the challenge of treating distal metastases. 3) Intracavitary Perfusion: OVs are administered into cavities such as the peritoneal, pleural, or bladder cavities. They can either diffuse directly to tumors within the cavity or be absorbed into the bloodstream to target tumor lesions. Intracavitary perfusion offers faster absorption compared to subcutaneous injection but slower absorption than intravenous administration. It is relatively simple to perform and requires less technical expertise, making it an ideal choice for targeting cavity-based organs (35). Current data indicate that intratumoral injection remains the most used delivery method for OVs (8). Intracavitary administration of oncolytic viruses is an intermediate form between intratumoral local administration and intravenous systemic administration. It can exert both direct tumor cell killing and modulation of immune cells to enhance anti-tumor immunity (36). Oncolytic virus intravenous systemic administration is one of the important methods of delivery and a potential direction for future development.

## 3 Challenges and advances in the management of malignant pleural effusion

### 3.1 The pathogenesis and composition of MPE

Malignant pleural effusion (MPE) refers to the accumulation of fluid between the lungs and the chest wall due to the presence and activity of cancer cells within the pleura. MPE is the second leading cause of exudative effusions (17) and represents a common complication of cancer, occurring in approximately 8–15% of cancer patients (18). MPE can be associated with almost any type of cancer. In men, most cases of MPE are caused by metastatic lung cancer, whereas in women, breast cancer metastases are the predominant cause. Together, lung and breast cancers account for 50–65% of all MPE cases. Lymphomas contribute to approximately 10% of MPE cases, while ovarian and gastric cancers account for around 5%. Malignant pleural mesothelioma is the most common primary pleural tumor, with over 90% of patients with malignant pleural mesothelioma presenting with MPE (19).

The presence of MPE is strongly associated with a poor quality of life due to symptoms such as dyspnea, pain, cachexia, fatigue, and reduced daily activity. Additionally, MPE is linked to a poor prognosis, with a median survival time of only 3–12 months. Current treatment strategies for MPE are primarily palliative and aim to alleviate symptoms (20, 21). MPE is typically composed of tumor cells, proteins, extracellular fluid, lymphocytes, and other metabolic products. Interactions between tumor cells and host immune cells create a specific immune microenvironment within the pleural cavity, favoring MPE formation. MPE microenvironment includes lymphocytes, particularly T cells (CD4^+^, CD8^+^), B cells, natural killer (NK) cells, and regulatory T cells (Tregs).

### 3.2 Current therapeutic approaches for MPE

#### 3.2.1 Thoracentesis

Thoracentesis is the initial treatment approach for MPE and is commonly performed to alleviate symptoms such as dyspnea and chest compression caused by unilateral or bilateral pleural effusion, pneumothorax, or pleural decompression. While thoracentesis provides symptom relief in most patients, its effects are generally transient, with recurrence typically occurring within one month. As a result, patients may require repeated procedures, with a maximum of 1.5 liters of fluid removed per session (37).

#### 3.2.2 Pleurodesis

Pleurodesis is a procedure aimed at obliterating the pleural space by inducing adhesion between the visceral and parietal pleura, thereby preventing the reaccumulation of fluid. It improves dyspnea, enhances survival rates (38), and reduces hospital stays and the need for future interventions (39–41). Although the optimal agent for pleurodesis remains undefined, talc is the most widely used due to its availability and cost-effectiveness. Talc can be administered via two methods: aerosolized talc insufflation through a thoracoscopic tube (talc poudrage) or as a suspension via an intercostal tube (talc slurry) (17). Other agents, such as antibiotics (tetracycline, doxycycline, and bleomycin), bacterial products (Bacillus Calmette–Guérin, OK432), silver nitrate, and povidone-iodine, have also been employed. A meta-analysis of 80 studies involving 5,507 patients demonstrated that talc is an effective pleurodesis agent with lower failure rates compared to bleomycin and doxycycline (42).

#### 3.2.3 Indwelling pleural catheters

IPCs are silicone tubes placed in the pleural cavity with a distal one-way valve and a subcutaneous tunnel. They enable outpatient fluid drainage, providing symptomatic relief by removing pleural effusion. Major guidelines recommend IPCs for symptomatic management of MPE, particularly in cases of trapped lung or failed prior pleurodesis. IPCs are now considered a first-line treatment option (43, 44). A TIME2 trial compared IPCs with chest tube drainage and talc pleurodesis for improving dyspnea and quality of life. The primary endpoint was the difference in dyspnea scores between the two groups at 42 days. The study found both methods equally effective in relieving dyspnea, with neither approach showing significant advantages in improving quality of life or dyspnea scores (45). The main drawback of IPCs is the risk of infection, including cellulitis, blockage, catheter dysfunction, pleural infection, and septated pleural effusion. The overall infection rate associated with IPCs is approximately 4.9%, with infection-related mortality at only 0.29% (46–48).

#### 3.2.4 Other therapeutic approaches

Current standard treatments for malignant pleural effusion (MPE) are predominantly palliative and include interventions such as thoracentesis, pleurodesis, and indwelling pleural catheters. However, the therapeutic efficacy of these approaches is limited, and patients often experience adverse effects such as chest pain and dyspnea. Thus, palliative interventions alone are insufficient to halt the progression of MPE, underscoring the importance of focusing on controlling the underlying malignancy. In recent years, advances in chemotherapy, targeted therapy, and immunotherapy have shown promise in managing cancers such as lung cancer, breast cancer, and lymphoma, which frequently lead to MPE. A phase II clinical trial evaluating the efficacy and safety of osimertinib combined with bevacizumab in patients with EGFR-mutant non-small cell lung cancer (NSCLC) with MPE demonstrated good safety but failed to significantly prolong progression-free survival (PFS) (49). Several phase III trials have shown that immune checkpoint inhibitors (ICIs) combined with chemotherapy significantly improve survival in advanced NSCLC patients with MPE compared to platinum-based doublet chemotherapy (50–53). Additionally, a retrospective study found that the combination of ICIs and chemotherapy significantly extended PFS compared to pembrolizumab monotherapy (54). For metastatic triple-negative breast cancer (mTNBC), a phase II study demonstrated that atezolizumab combined with paclitaxel and bevacizumab had tolerable safety (55). Another study showed that atezolizumab combined with nab-paclitaxel delayed disease progression without compromising patients’ quality of life (56). A randomized phase II trial indicated that lapatinib combined with trastuzumab was well-tolerated in HER2-positive breast cancer patients without chemotherapy (57). Intrathoracic drug delivery has also been reported for the treatment of MPE caused by solid tumors. A meta-analysis demonstrated that intrapleural cisplatin combined with low-dose interleukin-2 (IL-2) improved objective response rate (ORR), disease control rate (DCR), and quality of life (QOL) compared to cisplatin alone, without increasing the incidence of adverse events (AEs), apart from fever (58). A systematic review by Rong et al. revealed that Endostar combined with chemotherapy significantly improved ORR, DCR, and QOL compared to chemotherapy alone, without increasing the incidence of AEs (59). Nie et al. showed that intrapleural bevacizumab was more effective and safer than intravenous bevacizumab for NSCLC-related MPE (60). Wu et al. conducted a study where patients received intrapleural bevacizumab at three dose levels (2.5 mg/kg on days 1 and 8, 5 mg/kg on days 1 and 8, and 7.5 mg/kg on days 1 and 8). The ORR was 50%, and the PFS was 7.0 months, with the second dose group showing superior outcomes (61). Furthermore, a meta-analysis of intrapleural hyperthermic chemotherapy revealed a higher ORR for MPE patients without an increase in AEs (62). Anwarul et al. reported complete resolution of pleural effusion after four months of intrapleural rituximab in a patient with advanced low-grade B-cell lymphoma and MPE, with no recurrence for one year (63). Given the critical role of angiogenesis in MPE development, anti-angiogenic therapies have become a focus of treatment. Agents such as bevacizumab, apatinib, anlotinib, and recombinant human endostatin have shown promising results. Multiple studies have demonstrated that bevacizumab combined with chemotherapy is effective and well-tolerated in patients with lung cancer and MPE (64–66). Apatinib, a small-molecule tyrosine kinase inhibitor, selectively binds to vascular endothelial growth factor receptor 2 (VEGFR-2), strongly inhibiting its activity and reducing VEGF-mediated endothelial cell migration, proliferation, and tumor microvessel density (67). One study reported that apatinib combined with gemcitabine and cisplatin chemotherapy significantly improved DCR, ORR, tumor marker levels, and immune function in patients with advanced lung cancer and MPE. Anlotinib, a novel multi-target tyrosine kinase inhibitor, inhibits tumor angiogenesis and proliferation signaling (68). A phase II trial reported a DCR of 63.0% in small cell lung cancer (SCLC) patients treated with anlotinib, compared to 0% in the placebo group, with a median overall survival (OS) of 6.5 months versus 2.8 months in the placebo group (69).

## 4 Progress in oncolytic virus-based intrapleural therapy for MPE

Since systemic therapies are often ineffective for MPE due to limited access via the circulatory system, localized therapies such as intrapleural administration should be considered for MPE patients (70, 71). Intracavitary chemotherapy is a common treatment for MPE but often has low specificity for cancer cells, poor tumor localization, limited response rates, and significant side effects.

Common delivery methods for oncolytic viruses (OVs) include intratumoral and intravenous injections. However, intratumoral injection faces significant challenges for deep-seated tumors (72), while intravenous administration must overcome physiological barriers and neutralizing antibodies (73, 74). Intracavitary administration offers a localized delivery approach targeting body cavities, minimizing systemic toxicity. This method allows OVs to interact more directly with tumor cells and immune cells, facilitating direct tumor cell lysis and breaking immune tolerance. Intracavitary administration is primarily suitable for malignant pleural and peritoneal effusions, as well as primary and metastatic malignant tumors in the thoracic and abdominal cavities (75).

**Figure 1:**
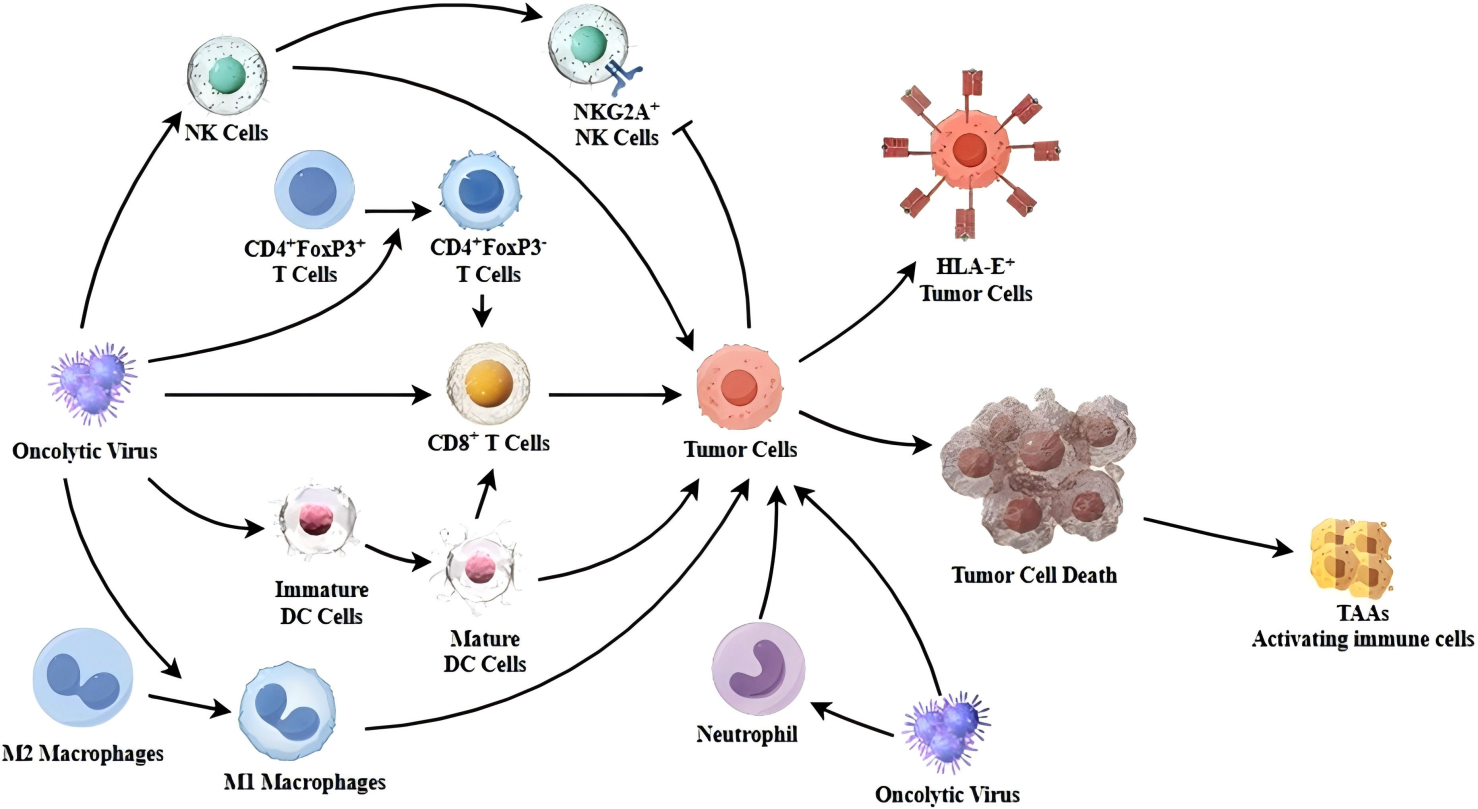
Mechanism of oncolytic viruses activating immune cells to exert antitumor immunity. OVs infect tumor cells, upregulating immune checkpoint molecules on tumor cells and releasing tumor-associated antigens (TAAs) upon tumor cell lysis. OVs reduce the number of FoxP3^+^CD4^+^ T cells in the tumor microenvironment and enhance the antitumor activity of CD8^+^ T cells. OVs promote the expression of immune checkpoint molecules on NK cells, enhance their antigen-presenting ability, and increase NK cell-mediated tumor cell killing. OVs stimulate the maturation of dendritic cells (DCs), leading to increased infiltration of CD8^+^ T cells at the tumor site. OVs induce the polarization of M2 macrophages into M1 macrophages, enhancing the antitumor immune response.

The treatment of MPE should meet the following conditions: killing tumor cells within the effusion, activating the antitumor immunity of lymphocytes in the effusion, and repairing damaged blood vessels and lymphatic vessels. Oncolytic viruses (OVs) have been reported to directly kill tumor cells, including inducing apoptosis, which is their fundamental antitumor mechanism (76). Oncolytic viruses can induce immunogenic cell death (ICD) in tumor cells, leading to increased release of tumor-associated antigens (TAAs), enhanced antigen presentation by antigen-presenting cells (APCs) (77, 78). Additionally, OVs can upregulate the expression of immune checkpoint molecules on both immune and tumor cells, including PD-1/PD-L1 and NKG2A/HLA-E (79, 80). This upregulation provides potential targets for subsequent combination therapy with immune checkpoint inhibitors (81, 82). Moreover, OVs can activate the antigen-presenting capacity of immune cells such as dendritic cells (DCs) and recruit T cells through the STING pathway (83–85), thereby enhancing T-cell-mediated antitumor responses (86, 87). Oncolytic virus can reduce the number of FoxP3^+^CD4^+^ T cells in the tumor microenvironment and facilitate the polarization of M2 macrophages into M1 macrophages (10, 88). Furthermore, OVs can recruit neutrophils and stimulate their antitumor activity (24) (**Figure 1**). Neutrophils have been shown to release reactive oxygen species (ROS) and neutrophil extracellular traps (NETs), which contribute to tumor cell killing. NETs are formed when neutrophils undergo cell death, releasing nuclear DNA and histones to create a highly adhesive web-like structure. Due to the high viscosity of DNA, these NETs serve as effective biological materials that adhere to the surface of damaged blood vessels, preventing fluid leakage and sealing off injured endothelial cells (25). Therefore, OVs exhibit multiple functions, including killing tumor cells, enhancing antitumor immune responses, and promoting vascular repair, all of which provide a strong foundation for the effective treatment of malignant pleural effusion (MPE).

Current clinical reports on the use of OVs for the treatment of MPE include:

H101 (Recombinant Adenovirus Type 5): A study involving 643 Chinese patients with MPE or malignant ascites showed an objective response rate (ORR) of 60.3%. In the monotherapy group, 60.4% achieved partial response (PR), with no significant differences between monotherapy and combination therapy groups. The main AEs were fever, nausea, and vomiting, with no severe events reported (89).

AdV-tk: A Phase I dose-escalation trial of gene-mediated cytotoxic immunotherapy (GMCI) with intrapleural AdV-tk combined with chemotherapy in MPE patients demonstrated safety and tolerability. Among 19 patients, 3 had prolonged stable disease. Notably, one patient survived 29 months after GMCI, showing significant efficacy (90).

ONCOS-102: In a randomized Phase I/II study for malignant pleural mesothelioma (MPM), ONCOS-102 combined with chemotherapy enhanced T-cell infiltration and upregulated immune response-related genes. Median OS was 20.3 months in first-line treatment patients compared to 13.5 months in the control group. Results support combining ONCOS-102 with immune checkpoint inhibitors (91).

HSV1716: A Phase I/IIa trial evaluated intrapleural HSV1716 in MPM patients. Thirteen patients received weekly injections, demonstrating good tolerability and minimal virus-related AEs. Viral replication was observed in 7 of 12 evaluable patients, and half the patients had stable disease at 8 weeks. Additionally, some patients developed novel tumor-specific IgG and Th1 cytokine responses (92).

## 5 Oncolytic virus therapy for intracavitary administration

In addition to intrapleural administration for the treatment of malignant pleural effusion, extensive research has been conducted on the intracavitary administration of oncolytic viruses (OVs) for other solid tumors. The underlying mechanism primarily involves using OVs to break local immune tolerance and activate antitumor immunity within the tumor microenvironment (75, 93). The immunosuppressive tumor microenvironment (TME) and poor immune infiltration are key reasons for the suboptimal efficacy of immunotherapy in solid tumors. The TME comprises tumor cells, vascular endothelial cells (ECs), cancer-associated fibroblasts (CAFs), and various resident or migratory immune cell subsets, such as T cells, dendritic cells (DCs), and natural killer (NK) cells (94). The immune suppression in TME arises from several mechanisms: 1) Tumor and stromal cells produce factors like transforming growth factor-β (TGF-β), prostaglandin E2 (PGE2), and interleukin-10 (IL-10), which impair the maturation of antigen-presenting cells (APCs) in the TME (95, 96). Consequently, DCs isolated from the TME often exhibit a partially mature, immunosuppressive phenotype. 2) Tumors suppress the production of T-cell-attracting chemokines CXCL9 and CXCL10, thereby reducing effector T-cell infiltration (95, 97). The effector T cells that infiltrate tumors are further weakened by prolonged antigen exposure and the expression of multiple immune checkpoint molecules. Thus, helper T cells and cytotoxic T lymphocytes (CTLs) isolated from the TME often display an exhausted phenotype. 3) Regulatory immune cells, such as CD4^+^ regulatory T cells (Tregs) and myeloid-derived suppressor cells (MDSCs), are recruited to tumor sites. Tregs secrete IL-10, indoleamine 2,3-dioxygenase (IDO), and TGF-β, which further suppress T-cell responses (95, 98). Additionally, Tregs consume IL-2, an essential cytokine for T-cell activation (95). MDSCs suppress effector T cells by producing arginase and nitric oxide, depriving T cells of amino acids required for proliferation (99, 100). Oncolytic viruses (OVs) promote tumor-specific T-cell recruitment and activation in the TME by mediating tumor cell lysis and inducing various forms of immunogenic cell death (ICD), including necrosis, necroptosis, pyroptosis, autophagic cell death, and immunogenic apoptosis (101, 102). OV infection of tumor cells triggers inflammation and local cytokine production, promoting the infiltration of innate immune cells and CTLs. This process helps reprogram the TME into a less immunosuppressive phenotype (100).

### 5.1 Bladder cancer

CG0070 is a replication-competent oncolytic adenovirus engineered to target RB-deficient tumor cells and express GM-CSF (103). A Phase II single-arm multicenter trial evaluated the 6-month efficacy of CG0070 in 45 patients, including 24 with carcinoma *in situ* (CIS), 8 with CIS and Ta tumors, 4 with CIS and T1 tumors, 6 with Ta tumors, and 3 with T1 tumors. The overall 6-month complete response (CR) rate was 47%, with 58% for CIS alone, 50% for CIS with Ta/T1 tumors, and 33% for Ta/T1 tumors alone. The only patient who progressed to muscle-invasive disease within 6 months had baseline Ta and T1 tumors. No patients with T1 tumors alone achieved CR at 6 months (14). Another study reported the safety and efficacy of CG0070 combined with nivolumab as neoadjuvant therapy in cisplatin-ineligible patients with muscle-invasive bladder cancer (MIBC). Among 21 enrolled patients, 15 were evaluable, and 8 (53%) achieved CR (104).

Cretostimogene grenadenorepvec is an oncolytic adenovirus type 5 that selectively replicates in cancer cells with abnormal RB pathways. Previously, it was used as monotherapy in non-muscle-invasive bladder cancer (NMIBC) patients who had failed bacillus Calmette-Guérin (BCG) therapy. A Phase II trial evaluated intravesical Cretostimogene combined with systemic pembrolizumab in BCG-unresponsive NMIBC patients with CIS. Among 35 treated patients, 82.9% achieved CR at 3 months. With a median follow-up of 26.5 months, the CR rate was 57.1% at 12 months and 51.4% at 24 months. No patients progressed to muscle-invasive disease. Adverse events (AEs) related to Cretostimogene were low-grade, self-limiting, and primarily bladder-related. Among 35 patients, 5 (14.3%) experienced grade 3 treatment-related AEs (15).

Nadofaragene firadenovec (nadofaragene firadenovec-vncg; Adstiladrin^®^) is a non-replicating adenoviral vector-based gene therapy developed by Ferring Pharmaceuticals. Adstiladrin^®^ contains vector DNA encoding interferon-alpha2b (IFN-α2b) and is the first gene therapy approved for bladder cancer treatment (105). A study by Stephen et al. reported the efficacy of intravesical nadofaragene firadenovec in BCG-unresponsive NMIBC patients. Among 157 enrolled patients, 151 were analyzable. Of the 103 patients with CIS (with or without high-grade Ta/T1 tumors), 55 (53.4%) achieved CR within 3 months of the first dose, and 25 (45.5%) of these 55 maintained CR at 12 months. The most common grade 3–4 AE related to the therapy was urinary urgency (2 patients, both grade 3). No treatment-related deaths occurred (106).

### 5.2 Ovarian cancer

Olvimulogene nanivacirepvec (Olvi-Vec; also known as GL-ONC1; laboratory name: GLV-1h68) is a modified oncolytic vaccinia virus engineered by inserting three expression cassettes encoding a Renilla luciferase-GFP fusion protein, β-galactosidase, and β-glucuronidase into the F14.5L, J2R, and A56R loci, respectively (107, 108). A study by Robert et al. evaluated the clinical activity of Olvi-Vec oncolytic immunotherapy combined with or without bevacizumab, followed by platinum-based doublet chemotherapy, in women with platinum-resistant or refractory ovarian cancer (PROC). Among 27 enrolled patients with platinum-resistant ovarian cancer (median of four prior treatment lines), 24 evaluable patients achieved an objective response rate of 54%, with a progression-free survival (PFS) of 11.0 months and manageable safety (16).

### 5.3 Peritoneal cancer (peritoneal mesothelioma)

A Phase I study evaluated the safety, maximum tolerated dose (MTD), and antitumor activity of intraperitoneal injection of GL-ONC1 in patients with advanced peritoneal cancer. Nine patients (seven with advanced peritoneal cancer and two with advanced peritoneal mesothelioma) received 24 doses of GL-ONC1. Adverse events (AEs) were limited to grades 1–3, including transient flu-like symptoms and treatment-induced peritonitis causing increased abdominal pain. No dose-limiting toxicities (DLTs) were reported, and the MTD was not reached. Eight out of nine patients demonstrated effective intraperitoneal infection, replication of GL-ONC1, and oncolytic activity during the first cycle. All patients developed neutralizing activity against GL-ONC1 (109).

## 6 Intratumoral administration of oncolytic viruses in solid tumors

Both the approved and commercially available oncolytic viruses, T-VEC and G47Δ (9, 10), adopt the method of intratumoral administration. Intratumoral administration is a common and effective treatment approach in oncolytic virus therapy and has demonstrated favorable therapeutic effects in the treatment of a variety of solid tumors (110, 111). After intratumoral administration for the treatment of multiple solid tumors, there have been observations such as the reduction in the size of tumors at the injection site, the decrease in the size of tumors at distant non-injection sites, and the extension of patients’ survival periods (11, 12). These results indicate that in addition to directly killing tumor cells, OVs can stimulate antitumor immune responses by activating immune cells within the tumor microenvironment (**Table 1**).

**Table 1:** Different types of OV and clinical applications.

Name of viral vector	Clinical indications	Name of Oncolytic Virus	Route of Administration
Reovirus	Malignant gliomas, ovarian cancer, and pancreatic cancer, etc.	Reolysin	Intravenous injection
Coxsackie virus	Melanoma	Coxsackievirus A21	Intratumoral injection
Adenovirus	Glioblastoma	Delta-24-RGD	Intratumoral injection
	Bladder cancer	CG0070	intrapleural administration
Vaccinia virus (VV)	Hepatocellular carcinoma	JX-594	Intratumoral injection
	Soft tissue sarcoma		Intravenous injection
Herpes simplex virus (HSV)	Melanoma	T-VEC	Intratumoral injection
		RP1	Intratumoral injection
	Glioblastoma	G47Δ	Intratumoral injection
		CAN-3110	Intratumoral injection
	Melanoma, sarcoma, and tumors of the digestive system	OH2	Intratumoral injection

### 6.1 Melanoma

#### 6.1.1 Talimogene laherparepvec

T-VEC, derived from herpes simplex virus type 1 (HSV-1), has been genetically modified to express granulocyte-macrophage colony-stimulating factor (GM-CSF), enhancing local immune responses against tumors. It is the first FDA-approved OV for treating advanced melanoma (112, 113). T-VEC demonstrates good tolerability and outperforms GM-CSF monotherapy, particularly in untreated patients or those with stage IIIB, IIIC, or IVM1a disease, achieving an overall response rate (ORR) of 31.5% and overall survival (OS) of 23.3 months versus 18.9 months (9, 11, 112, 114). Further studies explored combining T-VEC with immune checkpoint inhibitors (ICIs) like ipilimumab and pembrolizumab to enhance efficacy. Chesney et al. conducted a randomized trial showing that T-VEC combined with ipilimumab achieved a significantly higher ORR than ipilimumab monotherapy, with enhanced antitumor activity and no additional safety concerns (115). Antoni et al. reported that T-VEC therapy could improve PD-1 antibody (pembrolizumab) efficacy by altering the TME (76).

#### 6.1.2 CAVATAK

CAVATAK, derived from coxsackievirus A21, targets tumor cells with high expression of intercellular adhesion molecule-1 (ICAM-1), commonly found on melanoma cells. By infecting these tumor cells, CAVATAK induces oncolysis and an inflammatory response, attracting immune cell infiltration and stimulating antitumor immunity (116). In a phase II study of 57 patients with unresectable stage IIIC or IV melanoma, the ORR was 28.1%, with a durable response rate of 19.3% lasting ≥6 months. Median response time was 2.8 months, and the 1-year survival rate was 75.4%. CAVATAK demonstrated good tolerability and sustained local and systemic antitumor responses (117).

#### 6.1.3 RP1

RP1, a modified HSV-1, expresses GALV-GP R- fusion glycoprotein and GM-CSF to recruit and activate antitumor immune cells. Mohammed M. et al. reported that 36.1% of melanoma patients achieved partial (PR) or complete response (CR). Among patients who had not received prior anti-PD-1 therapy, 62.5% achieved the best response, compared to 37.5% among those who failed anti-PD-1/anti-PD-1+CTLA-4 therapy. RP1 was well-tolerated, with no new safety concerns (118).

#### 6.1.4 OrienX010

OrienX010, developed by OrienGene Biotechnology (China), is an HSV-1-based OV expressing GM-CSF (119). Cui et al. reported its safety and efficacy in unresectable stage IIIC-IV melanoma patients. Only one patient experienced grade ≥3 adverse events, and no dose-limiting toxicity (DLT) was observed. ORR was 19.2%, disease control rate (DCR) was 53.8%, and median duration of response (mDOR) was 6.0 months. Antitumor effects were observed in 54.6% of injected lesions and 48.8% of non-injected metastases. Median progression-free survival (PFS) and OS were 2.9 and 19.2 months, respectively (120).

#### 6.1.5 HF10

HF10 is a naturally occurring HSV-1 with unique genomic mutations (121). Robert et al. demonstrated that HF10 combined with ipilimumab showed both local and systemic antitumor activity, significantly improving response rates over ipilimumab monotherapy. At 24 weeks, the ORR was 37.8%, and DCR was 56.8% (122).

### 6.2 Lung cancer

A Phase II trial investigated the efficacy of intratumoral injection of oncolytic virus ADV/HSV-tk followed by stereotactic body radiotherapy (SBRT) at the same tumor site in patients with stage IV non-small cell lung cancer (NSCLC), including those who were treatment-naïve or resistant to prior PD-1 therapy. Among PD-1 therapy-naïve patients, the objective response rate (ORR) was 28.5%, and the clinical benefit rate (CBR) was 61.9%. For patients with prior immune checkpoint inhibitor (ICI) treatment, the ORR and CBR were 14.2% and 64.2%, respectively. This combination therapy was shown to restore sensitivity to ICIs in previously treated patients and benefit some tumors that lacked PD-L1 expression (123).

MEM-288 is a conditionally replicating oncolytic adenovirus with deletions in the E1A, E1B, and E3 regions of the viral genome. It expresses human interferon-beta (IFNβ) and a recombinant membrane-stable tumor necrosis factor-associated activation protein (TRAP) CD40L (124, 125). In a Phase I trial involving patients with refractory solid tumors, including 11 NSCLC patients, tumor shrinkage was observed at the injection site in 4 of 10 evaluable patients, with stabilization or shrinkage of distal non-injected lesions in several cases (126).

CAN-2409 is a non-replicating adenovirus serotype 5 expressing the herpes simplex virus thymidine kinase (HSV-TK) gene (127). A study evaluated its efficacy in patients with stage III/IV NSCLC who were non-responders to ICIs. Patients were treated with CAN-2409 in combination with valacyclovir while continuing ICI therapy. Among 73 treated patients, the median overall survival (mOS) was 22.0 months. Systemic clinical responses were observed in 64% of evaluable patients, with tumor shrinkage in both injected and non-injected lesions (128).

### 6.3 Gastrointestinal cancer

Oncorine (H101) is an oncolytic adenovirus derived from serotype 5, with deletions in the E1B-55k gene and four regions of the E3 gene. These modifications ensure selective replication in p53-deficient tumor cells while maintaining safety (129, 130). A retrospective analysis of 95 patients compared outcomes among three groups: H101 treatment alone, chemotherapy alone, and H101 combined with chemotherapy. The disease control rate (DCR) and ORR in the combined treatment group were 81.3% and 50.0%, respectively, significantly higher than those in the H101-only group (63.3% and 30.0%) and the chemotherapy-only group (66.7% and 33.3%). Additionally, the combined therapy group demonstrated superior 1-year and 2-year survival rates and progression-free survival (PFS) (131).

OH2 is a novel oncolytic herpes simplex virus (HSV) type II engineered to express human granulocyte-macrophage colony-stimulating factor (hGM-CSF) and to lack the ICP34.5 and ICP47 genes (132). Zhang et al. reported the results of a study evaluating OH2 as a monotherapy and in combination with the anti-PD-L1 antibody HX008 in patients with advanced solid tumors. Among 54 patients, including 18 with colorectal cancer, four patients achieved an immune partial response. Biopsy results after treatment revealed that OH2 modulates the tumor microenvironment (TME). Intratumoral injection of OH2 was well-tolerated and showed durable antitumor activity in colorectal cancer patients (133).

### 6.4 Hepatocellular carcinoma

VG161 is a type I oncolytic HSV that carries genes encoding interleukin (IL)-12, IL-15, the IL-15 receptor alpha subunit isoform 1 (IL-15RA), and a fusion protein (TF-Fc) that blocks PD-1/PD-L1 interactions. It also has deletions in the ICP34.5 gene to mitigate neurotoxicity (134). Shen et al. conducted a Phase I clinical trial in HCC patients who had failed two prior lines of therapy. The ORR was 17.14%, and the DCR was 60.00%, with a median PFS of 2.9 months and a median OS of 9.4 months. A significant OS benefit was observed in HCC subgroups, particularly in patients with prior treatment failure or specific genetic profiles. VG161 received breakthrough therapy designation from China’s National Medical Products Administration (NMPA) and became the first oncolytic virus product approved for HCC patients who had failed standard therapy (135).

### 6.5 Breast cancer

Pelareorep is an unencapsulated double-stranded RNA (dsRNA) virus with oncolytic activity capable of targeting multiple cancer cell types (136, 137). A randomized Phase II trial in HR+/HER2- metastatic breast cancer patients included 48 participants assigned to three treatment arms: paclitaxel (PTX) alone, PTX plus pelareorep, and PTX plus pelareorep with avelumab. At week 16, the ORRs were 20%, 31.3%, and 17.6%, respectively, while the DCRs were 46.7%, 62.5%, and 70.6%. Median PFS was 6.4 months, 9.6 months, and 7.5 months, respectively. The addition of pelareorep to PTX extended survival significantly; however, adding avelumab to the combination did not enhance efficacy (138).

Hatem et al. reported results from a Phase II trial investigating **T-VEC** in combination with neoadjuvant chemotherapy (NAC) for non-metastatic triple-negative breast cancer (TNBC). Among 37 evaluated patients, the residual cancer burden (RCB) 0 rate was 45.9%, and the RCB 0–1 rate was 65%. Two-year disease-free survival was 89%, with no recurrences observed in RCB 0–1 patient (139).

### 6.6 Brain tumors

G47Δ is a third-generation oncolytic herpes simplex virus type 1 (HSV-1) engineered with triple mutations. It was constructed by deleting the α47 gene and the overlapping US11 promoter from its parent virus, G207. Compared to G207, G47Δ demonstrates enhanced tumor-specific replication and cytopathic effects while maintaining high safety levels (140–142). In a Phase II single-arm trial, G47Δ was evaluated in 19 adult patients with supratentorial glioblastoma who had residual or recurrent disease following radiotherapy and temozolomide treatment. The 1-year survival rate after G47Δ administration was 84.2%, with an overall survival (OS) of 20.2 months and an OS of 28.8 months from the initial surgery. Magnetic resonance imaging (MRI) revealed repeated enlargement of the target lesion followed by clearance of contrast enhancement after each G47Δ administration. These findings highlight the survival benefits and favorable safety profile of G47Δ, which led to its approval as the first oncolytic virus product in Japan (10).

DNX-2401 (Delta-24-RGD, tasadenoturev) is an oncolytic adenovirus designed for tumor selectivity, enhanced infectivity, and replication capability. Tumor selectivity is achieved by a 24-base pair deletion in the E1A gene, preventing replication in normal cells with functional Rb pathways but allowing full replication in tumor cells (72). In a study of 49 patients with recurrent glioblastoma, intratumoral administration of DNX-2401 combined with intravenous pembrolizumab (an anti-PD-1 antibody) was evaluated. The objective response rate (ORR) was 10.4%, with a 12-month OS of 52.7% and a median OS of 12.5 months. Patients who achieved an objective response demonstrated longer survival, and 56.2% of patients experienced clinical benefit. Overall, intratumoral DNX-2401 combined with pembrolizumab was safe and provided significant survival benefits in select patients (143).

CAN-3110 retains the viral neurovirulence gene ICP34.5 under the control of the nestin promoter. Nestin is overexpressed in glioblastoma (GBM) and other aggressive tumors but is not expressed in adult brains or healthy differentiated tissues. These modifications enable CAN-3110 to preferentially replicate in tumor cells (144). Clinical data from the first-in-human trial of CAN-3110 in recurrent glioblastoma, reported by Alexander et al., demonstrated that intratumoral oncolytic virus therapy can enhance antitumor immune responses even within the immunosuppressive tumor microenvironment. This approach also provides a biological rationale for treating tumors resistant to other immunotherapies (145).

PVSRIPO is a non-neurotoxic chimera of rhinovirus and poliovirus that enters cells via the poliovirus receptor CD155, expressed on tumor cells and antigen-presenting cells. It promotes antitumor immune responses (146–148). A study evaluating PVSRIPO in recurrent glioblastoma reported that patients reached an OS plateau beginning at 24 months, with 24-month and 36-month OS rates of 21%. In contrast, historical controls showed continued declines, with OS rates of 14% at 24 months and 4% at 36 months (149).

## 7 Regulatory hurdles, cost impacts, and safety concerns for oncolytic virus treatment

### 7.1 Regulatory hurdles

#### 7.1.1 Virus spread and potential infection risk

Although oncolytic viruses are typically genetically modified to reduce toxicity, the risks of *in vivo* spread and latent infections still require long-term monitoring (150). Clinical trials of oncolytic viruses require testing of samples, such as swabs from injection sites, blood, and urine, for viral nucleic acids and TCID50 (74). However, detecting viral nucleic acids does not necessarily indicate the presence of live viruses (151). Moreover, the sensitivity of TCID50 testing is lower than that of nucleic acid detection methods (typically qPCR) (152). Therefore, establishing detection methods with higher sensitivity for live viruses could help reduce the potential spread risk during clinical treatment (153). Additionally, regarding latent infections, preclinical safety evaluations should assess whether the administration of oncolytic viruses could enhance the toxicity of latent wild-type viruses in the body.

#### 7.1.2 Tolerance and immune response

Due to differences in oncolytic virus vectors, their immunogenicity can lead to the generation of neutralizing antibodies after repeated administration, potentially affecting subsequent treatments (154, 155). Therefore, long-term follow-up is necessary to monitor virus tolerance and immune memory effects.

#### 7.1.3 Clinical trial design and endpoint determination

The unique mechanism of action of oncolytic viruses (OVs) presents significant regulatory challenges in endpoint determination. OVs not only exert direct antitumor effects by lysing tumor cells but also activate the immune system, leading to an abscopal effect that targets distant, non-injected tumor sites (156, 157). Tumor responses following OV treatment can be complex, as newly emerging lesions may signify either disease progression or delayed responses caused by treatment-induced inflammation or immune activation. This complexity makes it difficult to assess treatment outcomes using traditional criteria (158). The RECIST 1.1 guidelines, commonly used for evaluating solid tumor responses, are inadequate for capturing the full therapeutic potential of OVs, necessitating the adoption of immune-related response criteria (irRECIST) and other specialized evaluation frameworks that account for immune-mediated effects (159).

### 7.2 Cost implications

Compared to cell therapies, particularly chimeric antigen receptor T-cell (CAR-T) therapy, oncolytic viruses (OVs) can be produced on a large-scale using bioreactors, making their production costs relatively lower (160, 161). However, several challenges remain in the chemistry, manufacturing, and controls (CMC) process, including the stability of viral titers in each batch, the stability of expressed transgenes (especially when multiple genes are inserted), and issues related to host cell DNA and protein residues (162, 163). In terms of cost, compared to intratumoral administration, intrapleural administration of OV therapy does not significantly increase the required viral dose, thus avoiding a substantial rise in production costs (90, 123). However, differences in the dosing requirements of various OVs lead to variations in production costs (164). This is particularly evident in cases where the harvest fluid must be concentrated during the production process to obtain the final OV product, which increases the complexity and cost of impurity control (165, 166).

### 7.3 Accessibility of OV therapy

The accessibility of oncolytic virus (OV) therapy involves limitations in administration routes, challenges in virus manufacturing processes, and a limited range of approved indications.

Currently, OV therapy mainly relies on intratumoral injection, which is effective for tumors that are easily accessible or can be injected under image guidance (13). However, this method has limited efficacy for deep-seated tumors, restricting the widespread application of OVs in treating a broad range of solid tumors (167). Therefore, systemic intravenous (IV) administration or intracavitary administration has become a critical direction to enhance the accessibility of OVs. Despite its potential to target distant metastases, IV administration still faces challenges such as rapid neutralization by the host immune system (168), potential liver toxicity associated with high viral loads (169), limited tumor specificity (170), reducing therapeutic efficacy. Intracavitary administration, while providing a transition between intratumoral and systemic administration, it encounters challenges due to the complex intracavitary environment, including the presence of cellular debris (171), plasma proteins, and fibrin (172), which hinder the uniform diffusion of OVs and reduce viral infection efficiency within the cavity.

The manufacturing and purification processes for different OV platforms vary significantly, leading to differences in host cell selection (Vero (173), HEK293 (174), BHK-21 (175) cell lines are used depending on the virus platform), culturing methods (adherent culture and suspension culture) (176, 177), chromatography column selection (ion exchange chromatography, affinity chromatography) (178, 179), virus concentration techniques (tangential flow filtration (TFF), PEG precipitation, and density gradient centrifugation) (180, 181), host DNA and protein removal (ensuring residual DNA and protein levels meet regulatory requirements) (182, 183). These differences create challenges in achieving consistent, high-yield, and high-purity virus production, impacting the scalability and accessibility of OVs.

The range of approved OV therapies is limited (184), with most OVs only approved for a narrow spectrum of tumor types (185). Expanding the indications of OV therapy to a broader range of cancers requires extensive clinical data to support safety and efficacy. Further clinical validation across multiple tumor types is essential to increase the accessibility and applicability of OVs in clinical practice.

To improve the accessibility of OV therapy, overcoming limitations in administration routes, optimizing virus manufacturing processes, and expanding indications through clinical validation are critical steps. Successfully addressing these challenges will enhance the clinical application of OVs and broaden their therapeutic potential for a wider range of cancers.

### 7.4 Safety

In the preclinical safety evaluation, long-term toxicity studies conducted on cynomolgus monkeys using the HSV2-based oncolytic virus OH2 (HSV2 knockout of ICP34.5 and ICP47, insertion of hGM-CSF) and the oncolytic virus oHSV2-PDL1/CD3-BsAb (which shares the same viral backbone as OH2 but inserts PD-L1/CD3 bispecific antibody) demonstrated good safety following multiple subcutaneous administrations (186, 187). Other oncolytic viruses with different vectors, including adenovirus (188), vaccinia virus (189), and M1 virus (190), also exhibited good preclinical safety.

In the clinical trials that have been conducted, oncolytic virus intratumoral administration showed good safety profiles. The oncolytic virus OH2 did not cause any grade 3 or higher adverse events in various solid tumors (133). Other oncolytic viruses also showed good safety, with most adverse events being grade 3 or lower. A few grade 4 adverse events were reported, including cellulitis, gastrointestinal issues, lymphocytopenia, leukopenia, brain edema, speech disorders, hemiplegia, and urinary urgency, but no deaths related to oncolytic virus therapy occurred (8).

Immunological toxicities reported with oncolytic viruses mainly included cytokine release syndrome (CRS) and viral infections in the body (191). In a study by Aggarwal et al., 3 patients briefly experienced CRS after administration, which resolved within 3 days. In subsequent trials, the team added celecoxib to reduce the incidence or severity of CRS (90).

## 8 Conclusion and perspectives

In conclusion, malignant pleural effusion (MPE) remains a severe complication of malignant tumors, affecting a significant number of patients worldwide, and is associated with high mortality. The most common cancers causing MPE are lung cancer, followed by breast cancer, lymphoma, gynecological malignancies, and mesothelioma (19). MPE is typically a late-stage manifestation of disease, leading to poor prognosis, with median survival ranging from 3 to 12 months depending on the underlying malignancy and risk stratification. Patients with small cell lung cancer (SCLC) accompanied by MPE have a worse prognosis compared to those without MPE. Lymphoma patients with MPE at diagnosis have a higher risk of disease recurrence after chemotherapy. Lung cancer patients generally have the shortest survival, while mesothelioma and hematologic malignancy patients tend to have the longest survival. Other factors influencing survival include the degree of tumor infiltration into the pleura, characteristics of pleural effusion, biomarkers, the malignancy’s response to systemic treatment, and the patient’s baseline functional status (20, 21).

Patients with MPE commonly present with dyspnea, as tumor cells spread to the pleura and grow on its surface, which impairs lymphatic drainage and causes atelectasis and fluid accumulation within the pleural cavity. Malignant cells also stimulate the release of cytokines and upregulate angiogenesis factors, such as vascular endothelial growth factor (VEGF), which alter the osmotic pressure and permeability of the pleura and vasculature, contributing to the formation of MPE. However, most current treatments for MPE are palliative, with limited effectiveness in halting the progression of MPE. Future treatment strategies should focus on controlling the underlying tumor itself.

Oncolytic viruses can directly lyse tumor cells and stimulate immune cells to mount an anti-tumor response. The most common route of OV administration is intratumoral injection, which has been shown to have good safety and efficacy in clinical trials conducted for various solid tumors. Additionally, combining OVs with chemotherapy, radiotherapy, and other immunotherapies has been proven to enhance anti-tumor activity (28). Intracavitary perfusion of OVs is an intermediate approach between intratumoral and systemic intravenous administration. Compared to intratumoral injection, intracavitary delivery allows for more uniform distribution of the virus, enabling contact with multiple tumor lesions, and the procedure is relatively simple. Compared to intravenous administration, intracavitary administration achieves higher local concentrations of the virus, thereby reducing the potential systemic adverse effects associated with intravenous delivery (13). However, the intracavitary environment is relatively complex. Cellular debris, fibrin, and plasma protein can hinder the direct interaction between the virus and both tumor cells and immune cells (171, 172). One potential approach involves isolating lymphocytes from malignant pleural effusion ex vivo and co-incubating them with OVs *in vitro* to activate their anti-tumor activity. Clinical trials using this approach are already underway (NCT 05565014).

In addition, systemic intravenous administration of OVs is another important area of OV research. To achieve effective systemic delivery, future research can focus on the following aspects: modifying the viral capsid and envelope to better evade the host’s antiviral immune response during multiple intravenous administrations, thus reducing the production of neutralizing antibodies and avoiding liver toxicity (192); genetic modifications to the virus genome to enhance tumor specificity, replication ability, and immune evasion (193); using delivery systems, such as nanoparticle carriers, to improve the stability of oncolytic viruses, ensuring that, at safe doses, the virus has sufficient titer to specifically target tumor cells (194).

## References

[1] Gujar S Bell J Diallo J-S. SnapShot: cancer immunotherapy with oncolytic viruses. Cell. (2019) 176:1240–1240.e1241. doi: 10.1016/j.cell.2019.01.051 30794777

[2] Goradel NH Baker AT Arashkia A Ebrahimi N Ghorghanlu S Negahdari B. Oncolytic virotherapy: Challenges and solutions. Curr Problems Cancer. (2021) 45:100639. doi: 10.1016/j.currproblcancer.2020.100639 32828575

[3] Wang X Shao X Gu L Jiang K Wang S Chen J. Targeting STAT3 enhances NDV-induced immunogenic cell death in prostate cancer cells. J Cell Mol Med. (2020) 24:4286–97. doi: 10.1111/jcmm.15089 PMC7171322 32100392

[4] Shao X Wang X Guo X Jiang K Ye T Chen J. STAT3 contributes to oncolytic newcastle disease virus-induced immunogenic cell death in melanoma cells. Front Oncol. (2019) 9:436. doi: 10.3389/fonc.2019.00436 31192135 PMC6548873

[5] Breitbach CJ Arulanandam R De Silva N Thorne SH Patt R Daneshmand M. Oncolytic vaccinia virus disrupts tumor-associated vasculature in humans. Cancer Res. (2013) 73:1265–75. doi: 10.1158/0008-5472.Can-12-2687 23393196

[6] Kohlhapp FJ Kaufman HL. Molecular pathways: mechanism of action for talimogene laherparepvec, a new oncolytic virus immunotherapy. Clin Cancer Res. (2016) 22:1048–54. doi: 10.1158/1078-0432.Ccr-15-2667 26719429

[7] El-Shemi AG Ashshi AM Na Y Li Y Basalamah M Al-Allaf FA. Combined therapy with oncolytic adenoviruses encoding TRAIL and IL-12 genes markedly suppressed human hepatocellular carcinoma both *in vitro* and in an orthotopic transplanted mouse model. J Exp Clin Cancer Res. (2016) 35:74. doi: 10.1186/s13046-016-0353-8 27154307 PMC4859966

[8] Macedo N Miller DM Haq R Kaufman HL. Clinical landscape of oncolytic virus research in 2020. J ImmunoTherapy Cancer. (2020) 8:e001486. doi: 10.1136/jitc-2020-001486 PMC7552841 33046622

[9] Andtbacka RH Kaufman HL Collichio F Amatruda T Senzer N Chesney J. Talimogene laherparepvec improves durable response rate in patients with advanced melanoma. J Clin Oncol. (2015) 33:2780–8. doi: 10.1200/jco.2014.58.3377 26014293

[10] Todo T Ito H Ino Y Ohtsu H Ota Y Shibahara J. Intratumoral oncolytic herpes virus G47Δ for residual or recurrent glioblastoma: a phase 2 trial. Nat Med. (2022) 28:1630–9. doi: 10.1038/s41591-022-01897-x PMC9388376 35864254

[11] Andtbacka RH Ross M Puzanov I Milhem M Collichio F Delman KA. Patterns of clinical response with talimogene laherparepvec (T-VEC) in patients with melanoma treated in the OPTiM phase III clinical trial. Ann Surg Oncol. (2016) 23:4169–77. doi: 10.1245/s10434-016-5286-0 PMC5090012 27342831

[12] Tomita K Yamashita M Ikegami K Shimizu Y Amino N Nakao S. Combination with oxaliplatin improves abscopal effect of oncolytic virotherapy through reorganization of intratumoral macrophages. Mol Ther. (2025) 33:401–14. doi: 10.1016/j.ymthe.2024.12.007 PMC11764124 39663702

[13] Chintala NK Choe JK McGee E Bellis R Saini JK Banerjee S. Correlative analysis from a phase I clinical trial of intrapleural administration of oncolytic vaccinia virus (Olvi-vec) in patients with Malignant pleural mesothelioma. Front Immunol. (2023) 14:1112960. doi: 10.3389/fimmu.2023.1112960 36875061 PMC9977791

[14] Packiam VT Lamm DL Barocas DA Trainer A Fand B Davis RL 3rd. An open label, single-arm, phase II multicenter study of the safety and efficacy of CG0070 oncolytic vector regimen in patients with BCG-unresponsive non-muscle-invasive bladder cancer: Interim results. Urol Oncol. (2018) 36:440–7. doi: 10.1016/j.urolonc.2017.07.005 28755959

[15] Li R Shah PH Stewart TF Nam JK Bivalacqua TJ Lamm DL. Oncolytic adenoviral therapy plus pembrolizumab in BCG-unresponsive non-muscle-invasive bladder cancer: the phase 2 CORE-001 trial. Nat Med. (2024) 30:2216–23. doi: 10.1038/s41591-024-03025-3 38844794

[16] Holloway RW Mendivil AA Kendrick JE Abaid LN Brown JV LeBlanc J. Clinical activity of olvimulogene nanivacirepvec-Primed immunochemotherapy in heavily pretreated patients with platinum-Resistant or platinum-Refractory ovarian cancer: the nonrandomized phase 2 VIRO-15 clinical trial. JAMA Oncol. (2023) 9:903–8. doi: 10.1001/jamaoncol.2023.1007 PMC10214174 37227734

[17] Feller-Kopman DJ Reddy CB DeCamp MM Diekemper RL Gould MK Henry T. Management of Malignant pleural effusions. An official ATS/STS/STR clinical practice guideline. Am J Respir Crit Care Med. (2018) 198:839–49. doi: 10.1164/rccm.201807-1415ST 30272503

[18] Zamboni MM da Silva CT Jr. Baretta R Cunha ET Cardoso GP. Important prognostic factors for survival in patients with Malignant pleural effusion. BMC Pulm Med. (2015) 15:29. doi: 10.1186/s12890-015-0025-z 25887349 PMC4379612

[19] Gayen S. Malignant pleural effusion: presentation, diagnosis, and management. Am J Med. (2022) 135:1188–92. doi: 10.1016/j.amjmed.2022.04.017 35576996

[20] Porcel JM Gasol A Bielsa S Civit C Light RW Salud A. Clinical features and survival of lung cancer patients with pleural effusions. Respirology. (2015) 20:654–9. doi: 10.1111/resp.12496 25706291

[21] Bibby AC Dorn P Psallidas I Porcel JM Janssen J Froudarakis M. ERS/EACTS statement on the management of Malignant pleural effusions. Eur Respir J. (2018) 52:1800349. doi: 10.1183/13993003.00349-2018 30054348

[22] Delaunay T Achard C Boisgerault N Grard M Petithomme T Chatelain C. Frequent homozygous deletions of type I interferon genes in pleural mesothelioma confer sensitivity to oncolytic measles virus. J Thorac Oncol. (2020) 15:827–42. doi: 10.1016/j.jtho.2019.12.128 31945495

[23] DePeaux K Gunn WG Rivadeneira DB Delgoffe GM. Treatment with oncolytic vaccinia virus infects tumor-infiltrating regulatory and exhausted T cells. J Immunother Cancer. (2024) 12:e009062. doi: 10.1136/jitc-2024-009062 39153823 PMC11331848

[24] Zhou D Zhang C Sun J Yuan M. Neutrophils in oncolytic virus immunotherapy. Front Immunol. (2024) 15:1490414. doi: 10.3389/fimmu.2024.1490414 39697335 PMC11652357

[25] Xu P Tang K Ma J Zhang H Wang D Zhu L. Chemotherapeutic tumor microparticles elicit a neutrophil response targeting Malignant pleural effusions. Cancer Immunol Res. (2020) 8:1193–205. doi: 10.1158/2326-6066.Cir-19-0789 32661094

[26] Kaufman HL Kohlhapp FJ Zloza A. Oncolytic viruses: a new class of immunotherapy drugs. Nat Rev Drug Discovery. (2015) 14:642–62. doi: 10.1038/nrd4663 PMC7097180 26323545

[27] Gujar S Pol JG Kim Y Lee PW Kroemer G. Antitumor benefits of antiviral immunity: an underappreciated aspect of oncolytic virotherapies. Trends Immunol. (2018) 39:209–21. doi: 10.1016/j.it.2017.11.006 29275092

[28] Zhang B Cheng P. Improving antitumor efficacy via combinatorial regimens of oncolytic virotherapy. Mol Cancer. (2020) 19:158. doi: 10.1186/s12943-020-01275-6 33172438 PMC7656670

[29] Harrington KJ Puzanov I Hecht JR Hodi FS Szabo Z Murugappan S. Clinical development of talimogene laherparepvec (T-VEC): a modified herpes simplex virus type-1-derived oncolytic immunotherapy. Expert Rev Anticancer Ther. (2015) 15:1389–403. doi: 10.1586/14737140.2015.1115725 26558498

[30] Zamarin D Ricca JM Sadekova S Oseledchyk A Yu Y Blumenschein WM. PD-L1 in tumor microenvironment mediates resistance to oncolytic immunotherapy. J Clin Invest. (2018) 128:1413–28. doi: 10.1172/jci98047 PMC5873884 29504948

[31] Golden EB Frances D Pellicciotta I Demaria S Helen Barcellos-Hoff M Formenti SC. Radiation fosters dose-dependent and chemotherapy-induced immunogenic cell death. Oncoimmunology. (2014) 3:e28518. doi: 10.4161/onci.28518 25071979 PMC4106151

[32] Ghiringhelli F Menard C Puig PE Ladoire S Roux S Martin F. Metronomic cyclophosphamide regimen selectively depletes CD4+CD25+ regulatory T cells and restores T and NK effector functions in end stage cancer patients. Cancer Immunol Immunother. (2007) 56:641–8. doi: 10.1007/s00262-006-0225-8 PMC11030569 16960692

[33] Vincent J Mignot G Chalmin F Ladoire S Bruchard M Chevriaux A. 5-Fluorouracil selectively kills tumor-associated myeloid-derived suppressor cells resulting in enhanced T cell-dependent antitumor immunity. Cancer Res. (2010) 70:3052–61. doi: 10.1158/0008-5472.Can-09-3690 20388795

[34] Wang Y Zhang H Zhou Q Xia W Zhao X Li L. VP5 protein of oncolytic herpes simplex virus type 2 induces apoptosis in A549 cells through TP53I3 protein. Virology. (2024) 595:110093. doi: 10.1016/j.virol.2024.110093 38692134

[35] Li L Liu S Han D Tang B Ma J. Delivery and biosafety of oncolytic virotherapy. Front Oncol. (2020) 10:475. doi: 10.3389/fonc.2020.00475 32373515 PMC7176816

[36] Cerullo V Pesonen S Diaconu I Escutenaire S Arstila PT Ugolini M. Oncolytic adenovirus coding for granulocyte macrophage colony-stimulating factor induces antitumoral immunity in cancer patients. Cancer Res. (2010) 70:4297–309. doi: 10.1158/0008-5472.Can-09-3567 20484030

[37] Jacobs B Sheikh G Youness HA Keddissi JI Abdo T. Diagnosis and management of Malignant pleural effusion: A decade in review. Diagnostics (Basel). (2022) 12:1016. doi: 10.3390/diagnostics12041016 35454064 PMC9030780

[38] Hassan M Mercer RM Maskell NA Asciak R McCracken DJ Bedawi EO. Survival in patients with Malignant pleural effusion undergoing talc pleurodesis. Lung Cancer. (2019) 137:14–8. doi: 10.1016/j.lungcan.2019.09.003 31521977

[39] Thomas R Fysh ETH Smith NA Lee P Kwan BCH Yap E. Effect of an indwelling pleural catheter vs talc pleurodesis on hospitalization days in patients with Malignant pleural effusion: the AMPLE randomized clinical trial. Jama. (2017) 318:1903–12. doi: 10.1001/jama.2017.17426 PMC5820726 29164255

[40] Boshuizen RC Vd Noort V Burgers JA Herder GJM Hashemi SMS Hiltermann TJN. A randomized controlled trial comparing indwelling pleural catheters with talc pleurodesis (NVALT-14). Lung Cancer. (2017) 108:9–14. doi: 10.1016/j.lungcan.2017.01.019 28625655

[41] Freeman RK Ascioti AJ Dake M Mahidhara RS. A propensity-matched comparison of pleurodesis or tunneled pleural catheter for heart failure patients with recurrent pleural effusion. Ann Thorac Surg. (2014) 97:1872–6. doi: 10.1016/j.athoracsur.2014.02.027 24726601

[42] Dipper A Jones HE Bhatnagar R Preston NJ Maskell N Clive A. Interventions for the management of Malignant pleural effusions: an updated network meta-analysis. Eur Respir Rev. (2021) 4:CD010529. doi: 10.1002/14651858.CD010529.pub3 PMC9488663 33952602

[43] Roberts ME Neville E Berrisford RG Antunes G Ali NJ. Management of a Malignant pleural effusion: British Thoracic Society Pleural Disease Guideline 2010. Thorax. (2010) 65 Suppl 2:ii32–40. doi: 10.1136/thx.2010.136994 20696691

[44] Simoff MJ Lally B Slade MG Goldberg WG Lee P Michaud GC. Symptom management in patients with lung cancer: Diagnosis and management of lung cancer, 3rd ed: American College of Chest Physicians evidence-based clinical practice guidelines. Chest. (2013) 143:e455S–97S. doi: 10.1378/chest.12-2366 23649452

[45] Davies HE Mishra EK Kahan BC Wrightson JM Stanton AE Guhan A. Effect of an indwelling pleural catheter vs chest tube and talc pleurodesis for relieving dyspnea in patients with Malignant pleural effusion: the TIME2 randomized controlled trial. Jama. (2012) 307:2383–9. doi: 10.1001/jama.2012.5535 22610520

[46] Miller RJ Chrissian AA Lee YCG Rahman NM Wahidi MM Tremblay A. AABIP evidence-informed guidelines and expert panel report for the management of indwelling pleural catheters. J Bronchology Interv Pulmonol. (2020) 27:229–45. doi: 10.1097/lbr.0000000000000707 32804745

[47] Sundaralingam A Bedawi EO Harriss EK Munavvar M Rahman NM. The frequency, risk factors, and management of complications from pleural procedures. Chest. (2022) 161:1407–25. doi: 10.1016/j.chest.2021.11.031 34896096

[48] Fysh ETH Tremblay A Feller-Kopman D Mishra EK Slade M Garske L. Clinical outcomes of indwelling pleural catheter-related pleural infections: an international multicenter study. Chest. (2013) 144:1597–602. doi: 10.1378/chest.12-3103 23828305

[49] Hibino M Hiranuma O Takemura Y Katayama Y Chihara Y Harada T. Osimertinib and bevacizumab cotreatment for untreated EGFR-mutated NSCLC with Malignant pleural or pericardial effusion (SPIRAL II): A single-arm, open-label, phase 2 clinical trial. JTO Clin Res Rep. (2022) 3:100424. doi: 10.1016/j.jtocrr.2022.100424 36438852 PMC9692038

[50] Gandhi L Rodríguez-Abreu D Gadgeel S Esteban E Felip E De Angelis F. Pembrolizumab plus chemotherapy in metastatic non-small-cell lung cancer. N Engl J Med. (2018) 378:2078–92. doi: 10.1056/NEJMoa1801005 29658856

[51] Socinski MA Jotte RM Cappuzzo F Orlandi F Stroyakovskiy D Nogami N. Atezolizumab for first-line treatment of metastatic nonsquamous NSCLC. N Engl J Med. (2018) 378:2288–301. doi: 10.1056/NEJMoa1716948 29863955

[52] West H McCleod M Hussein M Morabito A Rittmeyer A Conter HJ. Atezolizumab in combination with carboplatin plus nab-paclitaxel chemotherapy compared with chemotherapy alone as first-line treatment for metastatic non-squamous non-small-cell lung cancer (IMpower130): a multicentre, randomised, open-label, phase 3 trial. Lancet Oncol. (2019) 20:924–37. doi: 10.1016/s1470-2045(19)30167-6 31122901

[53] Kawachi H Tamiya M Tamiya A Ishii S Hirano K Matsumoto H. Association between metastatic sites and first-line pembrolizumab treatment outcome for advanced non-small cell lung cancer with high PD-L1 expression: a retrospective multicenter cohort study. Invest New Drugs. (2020) 38:211–8. doi: 10.1007/s10637-019-00882-5 31784866

[54] Kawachi H Tamiya M Taniguchi Y Yokoyama T Yokoe S Oya Y. Efficacy of immune checkpoint inhibitor with or without chemotherapy for nonsquamous NSCLC with Malignant pleural effusion: A retrospective multicenter cohort study. JTO Clin Res Rep. (2022) 3:100355. doi: 10.1016/j.jtocrr.2022.100355 35769388 PMC9234704

[55] Cortes M Salgado AC Murillo SM Blancas I Cortez P Plaza IC. Safety interim analysis (SIA) of atractib: A phase 2 trial of first-line (1L) atezolizumab (A) in combination with paclitaxel (P) and bevacizumab (B) in metastatic triple-negative breast cancer (mTNBC). J Clin Oncol. (2022) 40:1084–4. doi: 10.1200/JCO.2022.40.16_suppl.1084

[56] Adams S Diéras V Barrios CH Winer EP Schneeweiss A Iwata H. Patient-reported outcomes from the phase III IMpassion130 trial of atezolizumab plus nab-paclitaxel in metastatic triple-negative breast cancer. Ann Oncol. (2020) 31:582–9. doi: 10.1016/j.annonc.2020.02.003 32178964

[57] Rimawi MF Niravath P Wang T Rexer BN Forero A Wolff AC. TBCRC023: A randomized phase II neoadjuvant trial of lapatinib plus trastuzumab without chemotherapy for 12 versus 24 weeks in patients with HER2-positive breast cancer. Clin Cancer Res. (2020) 26:821–7. doi: 10.1158/1078-0432.Ccr-19-0851 31662331

[58] Han L Jiang Q Yao W Fu T Zeng Q. Thoracic injection of low-dose interleukin-2 as an adjuvant therapy improves the control of the Malignant pleural effusions: a systematic review and meta-analysis base on Chinese patients. BMC Cancer. (2018) 18:725. doi: 10.1186/s12885-018-4581-5 29980186 PMC6035446

[59] Biaoxue R Xiguang C Hua L Wenlong G Shuanying Y. Thoracic perfusion of recombinant human endostatin (Endostar) combined with chemotherapeutic agents versus chemotherapeutic agents alone for treating Malignant pleural effusions: a systematic evaluation and meta-analysis. BMC Cancer. (2016) 16:888. doi: 10.1186/s12885-016-2935-4 27842514 PMC5109813

[60] Nie K Zhang Z You Y Zhuang X Zhang C Ji Y. A randomized clinical study to compare intrapleural infusion with intravenous infusion of bevacizumab in the management of Malignant pleural effusion in patients with non-small-cell lung cancer. Thorac Cancer. (2020) 11:8–14. doi: 10.1111/1759-7714.13238 31726490 PMC6938744

[61] Di W Yue C Ziran Z Jie Z Jun N Ling D. A phase II study of bevacizumab in non-squamous, non-small-cell lung cancer patients with Malignant pleural effusion. Future Oncol. (2022) 18:669–77. doi: 10.2217/fon-2021-1035 35080187

[62] Pan X Hou Z Zhang T Ding Z Ye F Wang Z. Efficacy and safety of intrapleural perfusion with hyperthermic chemotherapy for Malignant pleural effusion: a meta-analysis. J Cardiothorac Surg. (2024) 19:278. doi: 10.1186/s13019-024-02751-6 38711077 PMC11075297

[63] Islam A Takita H. Malignant pleural effusion and advanced stage low-Grade non-Hodgkin’s lymphoma successfully treated with intrapleural instillation of rituximab. Blood. (2012) 120:4891. doi: 10.1182/blood.V120.21.4891.4891

[64] Tamiya M Tamiya A Yamadori T Nakao K Asami K Yasue T. Phase2 study of bevacizumab with carboplatin-paclitaxel for non-small cell lung cancer with Malignant pleural effusion. Med Oncol. (2013) 30:676. doi: 10.1007/s12032-013-0676-7 23925664

[65] Tao H Meng Q Li M Shi L Tang J Liu Z. Outcomes of bevacizumab combined with chemotherapy in lung adenocarcinoma-induced Malignant pleural effusion. Thorac Cancer. (2018) 9:298–304. doi: 10.1111/1759-7714.12582 29297985 PMC5792722

[66] Tamiya M Tamiya A Suzuki H Taniguchi Y Katayama K Minomo S. Phase 2 study of bevacizumab plus carboplatin/nab-paclitaxel followed by bevacizumab plus nab-paclitaxel for non-squamous non-small cell lung cancer with Malignant pleural effusion. Invest New Drugs. (2021) 39:1106–12. doi: 10.1007/s10637-021-01076-8 33544282

[67] Li J Qin S Xu J Xiong J Wu C Bai Y. Randomized, double-blind, placebo-controlled phase III trial of apatinib in patients with chemotherapy-refractory advanced or metastatic adenocarcinoma of the stomach or gastroesophageal junction. J Clin Oncol. (2016) 34:1448–54. doi: 10.1200/jco.2015.63.5995 26884585

[68] Han B Li K Wang Q Zhang L Shi J Wang Z. Effect of anlotinib as a third-line or further treatment on overall survival of patients with advanced non-small cell lung cancer: the ALTER 0303 phase 3 randomized clinical trial. JAMA Oncol. (2018) 4:1569–75. doi: 10.1001/jamaoncol.2018.3039 PMC6248083 30098152

[69] Liu Y Cheng Y Wang Q Li K Shi J Wu L. Effectiveness of anlotinib in patients with small-cell lung cancer and pleural effusion: Subgroup analysis from a randomized, multicenter, phase II study. Thorac Cancer. (2021) 12:3039–45. doi: 10.1111/1759-7714.14176 PMC8590889 34596367

[70] Roberts ME Rahman NM Maskell NA Bibby AC Blyth KG Corcoran JP. British Thoracic Society Guideline for pleural disease. Thorax. (2023) 78:s1–s42. doi: 10.1136/thorax-2022-219784 37433578

[71] Chiang KY Ho JC Chong P Tam TC Lam DC Ip MS. Role of early definitive management for newly diagnosed Malignant pleural effusion related to lung cancer. Respirology. (2020) 25:1167–73. doi: 10.1111/resp.13812 32249488

[72] Lang FF Conrad C Gomez-Manzano C Yung WKA Sawaya R Weinberg JS. Phase I study of DNX-2401 (Delta-24-RGD) oncolytic adenovirus: replication and immunotherapeutic effects in recurrent Malignant glioma. J Clin Oncol. (2018) 36:1419–27. doi: 10.1200/jco.2017.75.8219 PMC6075856 29432077

[73] Di Paolo NC Baldwin LK Irons EE Papayannopoulou T Tomlinson S Shayakhmetov DM. IL-1α and complement cooperate in triggering local neutrophilic inflammation in response to adenovirus and eliminating virus-containing cells. PloS Pathog. (2014) 10:e1004035. doi: 10.1371/journal.ppat.1004035 24651866 PMC3961377

[74] Heo J Reid T Ruo L Breitbach CJ Rose S Bloomston M. Randomized dose-finding clinical trial of oncolytic immunotherapeutic vaccinia JX-594 in liver cancer. Nat Med. (2013) 19:329–36. doi: 10.1038/nm.3089 PMC4268543 23396206

[75] Ramesh N Ge Y Ennist DL Zhu M Mina M Ganesh S. CG0070, a conditionally replicating granulocyte macrophage colony-stimulating factor–armed oncolytic adenovirus for the treatment of bladder cancer. Clin Cancer Res. (2006) 12:305–13. doi: 10.1158/1078-0432.Ccr-05-1059 16397056

[76] Ribas A Dummer R Puzanov I VanderWalde A Andtbacka RHI Michielin O. Oncolytic virotherapy promotes intratumoral T cell infiltration and improves anti-PD-1 immunotherapy. Cell. (2017) 170:1109–1119.e1110. doi: 10.1016/j.cell.2017.08.027 28886381 PMC8034392

[77] van Vloten JP Workenhe ST Wootton SK Mossman KL Bridle BW. Critical interactions between immunogenic cancer cell death, oncolytic viruses, and the immune system define the rational design of combination immunotherapies. J Immunol. (2018) 200:450–8. doi: 10.4049/jimmunol.1701021 29311387

[78] Chen C Park AK Monroy I Ren Y Kim SI Chaurasiya S. Using oncolytic virus to retask CD19-chimeric antigen receptor T cells for treatment of pancreatic cancer: toward a universal chimeric antigen receptor T-cell strategy for solid tumor. J Am Coll Surg. (2024) 238:436–47. doi: 10.1097/xcs.0000000000000964 38214445

[79] Qin Y Cui Q Sun G Chao J Wang C Chen X. Developing enhanced immunotherapy using NKG2A knockout human pluripotent stem cell-derived NK cells. Cell Rep. (2024) 43:114867. doi: 10.1016/j.celrep.2024.114867 39447568

[80] Wang Y Jin J Li Y Zhou Q Yao R Wu Z. NK cell tumor therapy modulated by UV-inactivated oncolytic herpes simplex virus type 2 and checkpoint inhibitors. Transl Res. (2022) 240:64–86. doi: 10.1016/j.trsl.2021.10.006 34757194

[81] Borst L van der Burg SH van Hall T. The NKG2A-HLA-E axis as a novel checkpoint in the tumor microenvironment. Clin Cancer Res. (2020) 26:5549–56. doi: 10.1158/1078-0432.Ccr-19-2095 32409305

[82] Liu Z Ravindranathan R Kalinski P Guo ZS Bartlett DL. Rational combination of oncolytic vaccinia virus and PD-L1 blockade works synergistically to enhance therapeutic efficacy. Nat Commun. (2017) 8:14754. doi: 10.1038/ncomms14754 28345650 PMC5378974

[83] Kim Y Clements DR Sterea AM Jang HW Gujar SA Lee PW. Dendritic cells in oncolytic virus-Based anti-Cancer therapy. Viruses. (2015) 7:6506–25. doi: 10.3390/v7122953 PMC4690876 26690204

[84] Rajwani J Vishnevskiy D Turk M Naumenko V Gafuik C Kim DS. VSV(ΔM51) drives CD8(+) T cell-mediated tumour regression through infection of both cancer and non-cancer cells. Nat Commun. (2024) 15:9933. doi: 10.1038/s41467-024-54111-6 39548070 PMC11567966

[85] Dan J Cai J Zhong Y Wang C Huang S Zeng Y. Oncolytic virus M1 functions as a bifunctional checkpoint inhibitor to enhance the antitumor activity of DC vaccine. Cell Rep Med. (2023) 4:101229. doi: 10.1016/j.xcrm.2023.101229 37820722 PMC10591054

[86] Bommareddy PK Zloza A Rabkin SD Kaufman HL. Oncolytic virus immunotherapy induces immunogenic cell death and overcomes STING deficiency in melanoma. Oncoimmunology. (2019) 8:1591875. doi: 10.1080/2162402x.2019.1591875 31143509 PMC6527276

[87] Fu R Qi R Xiong H Lei X Jiang Y He J. Combination therapy with oncolytic virus and T cells or mRNA vaccine amplifies antitumor effects. Signal Transduct Target Ther. (2024) 9:118. doi: 10.1038/s41392-024-01824-1 38702343 PMC11068743

[88] Kong D Yang Z Li G Wu Q Gu Z Wan D. SIRPα antibody combined with oncolytic virus OH2 protects against tumours by activating innate immunity and reprogramming the tumour immune microenvironment. BMC Med. (2022) 20:376. doi: 10.1186/s12916-022-02574-z 36310169 PMC9620659

[89] Wang B Zhong C Liao Z Wang H Cai X Zhang Y. Effectiveness and safety of human type 5 recombinant adenovirus (H101) in Malignant tumor with Malignant pleural effusion and ascites: A multicenter, observational, real-world study. Thorac Cancer. (2023) 14:3051–7. doi: 10.1111/1759-7714.15101 PMC10599969 37675621

[90] Aggarwal C Haas AR Metzger S Aguilar LK Aguilar-Cordova E Manzanera AG. Phase I study of intrapleural gene-mediated cytotoxic immunotherapy in patients with Malignant pleural effusion. Mol Ther. (2018) 26:1198–205. doi: 10.1016/j.ymthe.2018.02.015 PMC5993936 29550074

[91] Hansen TB Cedres Perez S Ricordel C Levitsky V Ottesen LH Paz-Ares LG. Granular analysis of individual immune-related gene expression in a randomized phase I/II study of the oncolytic adenovirus, ONCOS-102, in combination with pemetrexed/cisplatin (P/C) in patients (pts) with unresectable Malignant pleural mesothelioma (MPM). J Clin Oncol. (2023) 41:e20536–6. doi: 10.1200/JCO.2023.41.16_suppl.e20536

[92] Danson SJ Conner J Edwards JG Blyth KG Fisher PM Muthana M. Oncolytic herpesvirus therapy for mesothelioma – A phase I/IIa trial of intrapleural administration of HSV1716. Lung Cancer. (2020) 150:145–51. doi: 10.1016/j.immuni.2013.07.005 33160198

[93] Gujar S Pol JG Kroemer G. Heating it up: Oncolytic viruses make tumors ‘hot’ and suitable for checkpoint blockade immunotherapies. Oncoimmunology. (2018) 7:e1442169. doi: 10.1080/2162402x.2018.1442169 30221036 PMC6136862

[94] Achard C Surendran A Wedge ME Ungerechts G Bell J Ilkow CS. Lighting a fire in the tumor microenvironment using oncolytic immunotherapy. EBioMedicine. (2018) 31:17–24. doi: 10.1016/j.ebiom.2018.04.020 29724655 PMC6013846

[95] Motz GT Coukos G. Deciphering and reversing tumor immune suppression. Immunity. (2013) 39:61–73. doi: 10.1016/j.immuni.2013.07.005 23890064 PMC3782392

[96] van der Burg SH Arens R Ossendorp F van Hall T Melief CJ. Vaccines for established cancer: overcoming the challenges posed by immune evasion. Nat Rev Cancer. (2016) 16:219–33. doi: 10.1038/nrc.2016.16 26965076

[97] Pitt JM Marabelle A Eggermont A Soria JC Kroemer G Zitvogel L. Targeting the tumor microenvironment: removing obstruction to anticancer immune responses and immunotherapy. Ann Oncol. (2016) 27:1482–92. doi: 10.1093/annonc/mdw168 27069014

[98] Yu J Du W Yan F Wang Y Li H Cao S. Myeloid-derived suppressor cells suppress antitumor immune responses through IDO expression and correlate with lymph node metastasis in patients with breast cancer. J Immunol. (2013) 190:3783–97. doi: 10.4049/jimmunol.1201449 23440412

[99] Bronte V Serafini P Mazzoni A Segal DM Zanovello P. L-arginine metabolism in myeloid cells controls T-lymphocyte functions. Trends Immunol. (2003) 24:302–6. doi: 10.1016/s1471-4906(03)00132-7 12810105

[100] de Graaf JF de Vor L Fouchier RAM van den Hoogen BG. Armed oncolytic viruses: A kick-start for anti-tumor immunity. Cytokine Growth Factor Rev. (2018) 41:28–39. doi: 10.1016/j.cytogfr.2018.03.006 29576283 PMC7108398

[101] Bartlett DL Liu Z Sathaiah M Ravindranathan R Guo Z He Y. Oncolytic viruses as therapeutic cancer vaccines. Mol Cancer. (2013) 12:103. doi: 10.1186/1476-4598-12-103 24020520 PMC3847443

[102] Guo ZS Liu Z Bartlett DL. Oncolytic immunotherapy: dying the right way is a key to eliciting potent antitumor immunity. Front Oncol. (2014) 4:74. doi: 10.3389/fonc.2014.00074 24782985 PMC3989763

[103] Grandi P Darilek A Moscu A Pradhan A Li R. Intravesical infusion of oncolytic virus CG0070 in the treatment of bladder cancer. Methods Mol Biol. (2023) 2684:303–17. doi: 10.1007/978-1-0716-3291-8_19 37410243

[104] Kim H Jeong BC Hong J Kwon GY Kim CK Park W. Neoadjuvant nivolumab plus gemcitabine/cisplatin chemotherapy in muscle-invasive urothelial carcinoma of the bladder. Cancer Res Treat. (2023) 55:636–42. doi: 10.4143/crt.2022.343 PMC10101782 36228654

[105] Lee A. Nadofaragene firadenovec: first approval. Drugs. (2023) 83:353–7. doi: 10.1007/s40265-023-01846-z PMC10239371 36856952

[106] Boorjian SA Alemozaffar M Konety BR Shore ND Gomella LG Kamat AM. Intravesical nadofaragene firadenovec gene therapy for BCG-unresponsive non-muscle-invasive bladder cancer: a single-arm, open-label, repeat-dose clinical trial. Lancet Oncol. (2021) 22:107–17. doi: 10.1016/s1470-2045(20)30540-4 PMC7988888 33253641

[107] Zhang Q Yu YA Wang E Chen N Danner RL Munson PJ. Eradication of solid human breast tumors in nude mice with an intravenously injected light-emitting oncolytic vaccinia virus. Cancer Res. (2007) 67:10038–46. doi: 10.1158/0008-5472.Can-07-0146 17942938

[108] Ascierto ML Worschech A Yu Z Adams S Reinboth J Chen NG. Permissivity of the NCI-60 cancer cell lines to oncolytic Vaccinia Virus GLV-1h68. BMC Cancer. (2011) 11:451. doi: 10.1186/1471-2407-11-451 22011439 PMC3213037

[109] Lauer UM Schell M Beil J Berchtold S Koppenhöfer U Glatzle J. Phase I study of oncolytic vaccinia virus GL-ONC1 in patients with peritoneal carcinomatosis. Clin Cancer Res. (2018) 24:4388–98. doi: 10.1158/1078-0432.Ccr-18-0244 29773661

[110] Estrada J Zhan J Mitchell P Werner J Beltran PJ DeVoss J. OncoVEX(mGM-CSF)expands tumor antigen-specific CD8+ T-cell response in preclinical models. J Immunother Cancer. (2023) 11:e006374. doi: 10.1136/jitc-2022-006374 37164449 PMC10173969

[111] Shen Y Bai X Zhang Q Liang X Jin X Zhao Z. Oncolytic virus VG161 in refractory hepatocellular carcinoma. Nature. (2025). doi: 10.1038/s41586-025-08717-5 40108464

[112] Cohen S Boland GM. Harnessing the potential of combination immunotherapy and oncolytic virotherapy for solid tumors. Ann Surg Oncol. (2022) 29:762–3. doi: 10.1245/s10434-021-11059-x 34837138

[113] Conry RM Westbrook B McKee S Norwood TG. Talimogene laherparepvec: First in class oncolytic virotherapy. Hum Vaccin Immunother. (2018) 14:839–46. doi: 10.1080/21645515.2017.1412896 PMC5893211 29420123

[114] Andtbacka RHI Collichio F Harrington KJ Middleton MR Downey G Öhrling K. Final analyses of OPTiM: a randomized phase III trial of talimogene laherparepvec versus granulocyte-macrophage colony-stimulating factor in unresectable stage III-IV melanoma. J Immunother Cancer. (2019) 7:145. doi: 10.1186/s40425-019-0623-z 31171039 PMC6554874

[115] Chesney J Puzanov I Collichio F Singh P Milhem MM Glaspy J. Randomized, open-label phase II study evaluating the efficacy and safety of talimogene laherparepvec in combination with ipilimumab versus ipilimumab alone in patients with advanced, unresectable melanoma. J Clin Oncol. (2018) 36:1658–67. doi: 10.1200/jco.2017.73.7379 PMC6075852 28981385

[116] Bradley S Jakes AD Harrington K Pandha H Melcher A Errington-Mais F. Applications of coxsackievirus A21 in oncology. Oncolytic Virother. (2014) 3:47–55. doi: 10.2147/ov.S56322 27512662 PMC4918364

[117] Andtbacka RHI Curti B Daniels GA Hallmeyer S Whitman ED Lutzky J. Clinical responses of oncolytic coxsackievirus A21 (V937) in patients with unresectable melanoma. J Clin Oncol. (2021) 39:3829–38. doi: 10.1200/jco.20.03246 34464163

[118] Milhem MM Vanderwalde AM Bowles TL Sacco JJ Niu J Tsai KK. Updated results from the skin cancer cohorts from an ongoing phase 1/2 multicohort study of RP1, an enhanced potency oncolytic HSV, combined with nivolumab (IGNYTE). J Clin Oncol. (2022) 40:9553–3. doi: 10.1200/JCO.2022.40.16_suppl.9553

[119] Ren J Gwin WR Zhou X Wang X Huang H Jiang N. Adaptive T cell responses induced by oncolytic Herpes Simplex Virus-granulocyte macrophage-colony-stimulating factor therapy expanded by dendritic cell and cytokine-induced killer cell adoptive therapy. Oncoimmunology. (2017) 6:e1264563. doi: 10.1080/2162402x.2016.1264563 28507788 PMC5414875

[120] Cui C Wang X Lian B Ji Q Zhou L Chi Z. OrienX010, an oncolytic virus, in patients with unresectable stage IIIC-IV melanoma: a phase Ib study. J Immunother Cancer. (2022) 10:e004307. doi: 10.1136/jitc-2021-004307 35383116 PMC8984036

[121] Eissa IR Naoe Y Bustos-Villalobos I Ichinose T Tanaka M Zhiwen W. Genomic signature of the natural oncolytic herpes simplex virus HF10 and its therapeutic role in preclinical and clinical trials. Front Oncol. (2017) 7:149. doi: 10.3389/fonc.2017.00149 28770166 PMC5509757

[122] Andtbacka RHI Ross MI Agarwala SS Taylor MH Vetto JT Neves RI. Preliminary results from phase II study of combination treatment with HF10, a replication-competent HSV-1 oncolytic virus, and ipilimumab in patients with stage IIIb, IIIc, or IV unresectable or metastatic melanoma. J Clin Oncol. (2016) 34:9543–3. doi: 10.1200/JCO.2016.34.15_suppl.9543

[123] Guerrero C Ensor JE Sun K Farach AM Nair S Zhang J. Stereotactic body radiation therapy and in *situ* oncolytic virus therapy followed by immunotherapy in metastatic non-small cell lung cancer. J Clin Oncol. (2021) 39:9115–5. doi: 10.1200/JCO.2021.39.15_suppl.9115

[124] Zheng H Yu X Ibrahim ML Foresman D Xie M Johnson JO. Combination IFNβ and membrane-stable CD40L maximize tumor dendritic cell activation and lymph node trafficking to elicit systemic T-cell immunity. Cancer Immunol Res. (2023) 11:466–85. doi: 10.1158/2326-6066.Cir-22-0927 PMC10165690 36757308

[125] Peters PN Whitaker RS Lim F Russell S Bloom EA Pollara J. Oncolytic adenovirus MEM-288 encoding membrane-stable CD40L and IFNβ induces an anti-tumor immune response in high grade serous ovarian cancer. Neoplasia. (2024) 57:101056. doi: 10.1016/j.neo.2024.101056 39276533 PMC11417341

[126] Saltos AN Arrowood C Beasley G Ronald J El-Haddad G Guerra-Guevara L. A phase 1 first-in-human study of interferon beta (IFNβ) and membrane-stable CD40L expressing oncolytic virus (MEM-288) in solid tumors including non–small-cell lung cancer (NSCLC). J Clin Oncol. (2023) 41:2569–9. doi: 10.1200/JCO.2023.41.16_suppl.2569

[127] Koch MS Zdioruk M Nowicki MO Griffith AM Aguilar E Aguilar LK. Systemic high-dose dexamethasone treatment may modulate the efficacy of intratumoral viral oncolytic immunotherapy in glioblastoma models. J Immunother Cancer. (2022) 10:e003368. doi: 10.1136/jitc-2021-003368 35017150 PMC8753448

[128] Aggarwal C Sterman D Alesi ER Maldonado F Mehra R Bestvina CM. Overall survival after treatment with CAN-2409 plus valacyclovir in combination with continued ICI in patients with stage III/IV NSCLC with an inadequate response to ICI. J Clin Oncol. (2024) 42:8634–4. doi: 10.1200/JCO.2024.42.16_suppl.8634

[129] Liang M. Oncorine, the world first oncolytic virus medicine and its update in China. Curr Cancer Drug Targets. (2018) 18:171–6. doi: 10.2174/1568009618666171129221503 29189159

[130] Cheng G Dong H Yang C Liu Y Wu Y Zhu L. A review on the advances and challenges of immunotherapy for head and neck cancer. Cancer Cell Int. (2021) 21:406. doi: 10.1186/s12935-021-02024-5 34332576 PMC8325213

[131] Zhang R Cui Y Guan X Jiang X. A recombinant human adenovirus type 5 (H101) combined with chemotherapy for advanced gastric carcinoma: A retrospective cohort study. Front Oncol. (2021) 11:752504. doi: 10.3389/fonc.2021.752504 34956877 PMC8695551

[132] Zhao Q Zhang W Ning Z Zhuang X Lu H Liang J. A novel oncolytic herpes simplex virus type 2 has potent anti-tumor activity. PloS One. (2014) 9:e93103. doi: 10.1371/journal.pone.0093103 24671154 PMC3966855

[133] Zhang B Huang J Tang J Hu S Luo S Luo Z. Intratumoral OH2, an oncolytic herpes simplex virus 2, in patients with advanced solid tumors: a multicenter, phase I/II clinical trial. J Immunother Cancer. (2021) 9:e002224. doi: 10.1136/jitc-2020-002224 33837053 PMC8043042

[134] Shen Y Song W Lin D Zhang X Wang M Li Y. VG161 activates systemic antitumor immunity in pancreatic cancer models as a novel oncolytic herpesvirus expressing multiple immunomodulatory transgenes. J Med Virol. (2023) 95:e28108. doi: 10.1002/jmv.28108 36042555 PMC10087349

[135] Shen Y Liang X Jin X Tan Q Zhao R Wei G. Clinical outcomes from a phase I clinical trial of a novel oncolytic virus VG161 in patients with hepatocellular carcinoma (HCC) refractory after 2 prior lines of therapy including checkpoint inhibitors (CPI). J Clin Oncol. (2024) 42:4105–5. doi: 10.1200/JCO.2024.42.16_suppl.4105

[136] Clements D Helson E Gujar SA Lee PW. Reovirus in cancer therapy: an evidence-based review. Oncolytic Virother. (2014) 3:69–82. doi: 10.2147/ov.S51321 27512664 PMC4918368

[137] Lundstrom K. Viral vectors in gene therapy: where do we stand in 2023? Viruses. (2023) 15:698. doi: 10.3390/v15030698 36992407 PMC10059137

[138] Clark AS Zhao F Klein P Montero AJ Falkson CI Krill-Jackson E. BRACELET-1 (PrE0113): Inducing an inflammatory phenotype in metastatic HR+/HER2- breast cancer with the oncolytic reovirus pelareorep in combination with paclitaxel and avelumab. J Clin Oncol. (2023) 41:1012–2. doi: 10.1200/JCO.2023.41.16_suppl.1012

[139] Soliman H Hogue D Han H Mooney B Costa R Lee MC. Oncolytic T-VEC virotherapy plus neoadjuvant chemotherapy in nonmetastatic triple-negative breast cancer: a phase 2 trial. Nat Med. (2023) 29:450–7. doi: 10.1038/s41591-023-02210-0 36759673

[140] Todo T Martuza RL Rabkin SD Johnson PA. Oncolytic herpes simplex virus vector with enhanced MHC class I presentation and tumor cell killing. Proc Natl Acad Sci U.S.A. (2001) 98:6396–401. doi: 10.1073/pnas.101136398 PMC33479 11353831

[141] Ma W He H Wang H. Oncolytic herpes simplex virus and immunotherapy. BMC Immunol. (2018) 19:40. doi: 10.1186/s12865-018-0281-9 30563466 PMC6299639

[142] Carson J Haddad D Bressman M Fong Y. ONCOLYTIC HERPES SIMPLEX VIRUS 1 (HSV-1) VECTORS: INCREASING TREATMENT EFFICACY AND RANGE THROUGH STRATEGIC VIRUS DESIGN. Drugs Future. (2010) 35:183–95. doi: 10.1358/dof.2010.35.3.1470166 PMC3266817 22287818

[143] Nassiri F Patil V Yefet LS Singh O Liu J Dang RMA. Oncolytic DNX-2401 virotherapy plus pembrolizumab in recurrent glioblastoma: a phase 1/2 trial. Nat Med. (2023) 29:1370–8. doi: 10.1038/s41591-023-02347-y PMC10287560 37188783

[144] Chiocca EA Nakashima H Kasai K Fernandez SA Oglesbee M. Preclinical toxicology of rQNestin34.5v.2: an oncolytic herpes virus with transcriptional regulation of the ICP34.5 neurovirulence gene. Mol Ther Methods Clin Dev. (2020) 17:871–93. doi: 10.1016/j.omtm.2020.03.028 PMC7195500 32373649

[145] Ling AL Solomon IH Landivar AM Nakashima H Woods JK Santos A. Clinical trial links oncolytic immunoactivation to survival in glioblastoma. Nature. (2023) 623:157–66. doi: 10.1038/s41586-023-06623-2 PMC10620094 37853118

[146] Gromeier M Lachmann S Rosenfeld MR Gutin PH Wimmer E. Intergeneric poliovirus recombinants for the treatment of Malignant glioma. Proc Natl Acad Sci U.S.A. (2000) 97:6803–8. doi: 10.1073/pnas.97.12.6803 PMC18745 10841575

[147] Beasley GM Nair SK Farrow NE Landa K Selim MA Wiggs CA. Phase I trial of intratumoral PVSRIPO in patients with unresectable, treatment-refractory melanoma. J Immunother Cancer. (2021) 9:e002203. doi: 10.1136/jitc-2020-002203 33875611 PMC8057552

[148] Thompson EM Kang KD Stevenson K Zhang H Gromeier M Ashley D. Elucidating cellular response to treatment with viral immunotherapies in pediatric high-grade glioma and medulloblastoma. Transl Oncol. (2024) 40:101875. doi: 10.1016/j.tranon.2024.101875 38183802 PMC10809117

[149] Desjardins A Gromeier M Herndon JE 2nd Beaubier N Bolognesi DP Friedman AH. Recurrent glioblastoma treated with recombinant poliovirus. N Engl J Med. (2018) 379:150–61. doi: 10.1056/NEJMoa1716435 PMC6065102 29943666

[150] Pol JG Zhang L Bridle BW Stephenson KB Rességuier J Hanson S. Maraba virus as a potent oncolytic vaccine vector. Mol Ther. (2014) 22:420–9. doi: 10.1038/mt.2013.249 PMC3916044 24322333

[151] Laassri M Mee ET Connaughton SM Manukyan H Gruber M Rodriguez-Hernandez C. Detection of bovine viral diarrhoea virus nucleic acid, but not infectious virus, in bovine serum used for human vaccine manufacture. Biologicals. (2018) 55:63–70. doi: 10.1016/j.biologicals.2018.06.002 29941334

[152] Soni A Herbert C Lin H Yan Y Pretz C Stamegna P. Performance of rapid antigen tests to detect symptomatic and asymptomatic SARS-coV-2 infection: A prospective cohort study. Ann Intern Med. (2023) 176:975–82. doi: 10.7326/m23-0385 PMC10321467 37399548

[153] Guo M Deng L Liang H Du Y Gao W Tian N. Development and preliminary application of a droplet digital PCR assay for quantifying the oncolytic herpes simplex virus type 1 in the clinical-grade production. Viruses. (2023) 15:178. doi: 10.3390/v15010178 36680218 PMC9867280

[154] Shin DH Jiang H Gillard AG Kim D Fan X Singh SK. Chimeric oncolytic adenovirus evades neutralizing antibodies from human patients and exhibits enhanced anti-glioma efficacy in immunized mice. Mol Ther. (2024) 32:722–33. doi: 10.1016/j.ymthe.2024.01.035 PMC10928285 38311852

[155] Sitta J De Carlo F Kirven I Tackett JH Penfornis P Dobbins GC. Microbubble-protected oncolytic virotherapy targeted by sonoporation induces tumor necrosis and T-lymphocyte infiltration in humanized mice bearing triple-negative breast cancer. Int J Mol Sci. (2024) 25:13697. doi: 10.3390/ijms252413697 39769460 PMC11678396

[156] Zhang N Guan Y Li J Yu J Yi T. Inactivation of the DNA-sensing pathway facilitates oncolytic herpes simplex virus inhibition of pancreatic ductal adenocarcinoma growth. Int Immunopharmacol. (2023) 124:110969. doi: 10.1016/j.intimp.2023.110969 37774484

[157] Ju F Luo Y Lin C Jia X Xu Z Tian R. Oncolytic virus expressing PD-1 inhibitors activates a collaborative intratumoral immune response to control tumor and synergizes with CTLA-4 or TIM-3 blockade. J Immunother Cancer. (2022) 10:e004762. doi: 10.1136/jitc-2022-004762 35688558 PMC9189843

[158] Hales RK Banchereau J Ribas A Tarhini AA Weber JS Fox BA. Drake CG Assessing oncologic benefit in clinical trials of immunotherapy agents. Ann Oncol. (2010) 21:1944–51. doi: 10.1093/annonc/mdq048 20237004

[159] Manitz J D’Angelo SP Apolo AB Eggleton SP Bajars M Bohnsack O. Comparison of tumor assessments using RECIST 1.1 and irRECIST, and association with overall survival. J Immunother Cancer. (2022) 10:e003302. doi: 10.1136/jitc-2021-003302 35228264 PMC8886415

[160] Bulcha JT Wang Y Ma H Tai PWL Gao G. Viral vector platforms within the gene therapy landscape. Signal Transduct Target Ther. (2021) 6:53. doi: 10.1038/s41392-021-00487-6 33558455 PMC7868676

[161] Kaštánková I Štach M Žižková H Ptáčková P Šmilauerová K Mucha M. Enzymatically produced piggyBac transposon vectors for efficient non-viral manufacturing of CD19-specific CAR T cells. Mol Ther Methods Clin Dev. (2021) 23:119–27. doi: 10.1016/j.omtm.2021.08.006 PMC8482285 34631931

[162] Gimpel AL Katsikis G Sha S Maloney AJ Hong MS Nguyen TNT. Analytical methods for process and product characterization of recombinant adeno-associated virus-based gene therapies. Mol Ther Methods Clin Dev. (2021) 20:740–54. doi: 10.1016/j.omtm.2021.02.010 PMC7940698 33738328

[163] Wechman SL Rao XM Cheng PH Gomez-Gutierrez JG McMasters KM Zhou HS. Development of an oncolytic adenovirus with enhanced spread ability through repeated UV irradiation and cancer selection. Viruses. (2016) 8:167. doi: 10.3390/v8060167 27314377 PMC4926187

[164] Habisch R Neubauer P Soza-Ried J Puschmann E. Repeated harvest enables efficient production of VSV-GP. Front Bioeng Biotechnol. (2024) 12:1505338. doi: 10.3389/fbioe.2024.1505338 39703791 PMC11656157

[165] Clément N Grieger JC. Manufacturing of recombinant adeno-associated viral vectors for clinical trials. Mol Ther - Methods Clin Dev. (2016) 3:16002. doi: 10.1038/mtm.2016.2 27014711 PMC4804725

[166] Kimura T Ferran B Tsukahara Y Shang Q Desai S Fedoce A. Production of adeno-associated virus vectors for *in vitro* and *in vivo* applications. Sci Rep. (2019) 9:13601. doi: 10.1038/s41598-019-49624-w 31537820 PMC6753157

[167] Del Papa J Clarkin RG Parks RJ. Use of cell fusion proteins to enhance adenoviral vector efficacy as an anti-cancer therapeutic. Cancer Gene Ther. (2021) 28:745–56. doi: 10.1038/s41417-020-0192-9 32606392

[168] Tang G Wang D Zhao X Feng Z Chen Q Shen Y. The dilemma of HSV-1 oncolytic virus delivery: the method choice and hurdles. Int J Mol Sci. (2023) 24:3681. doi: 10.3390/ijms24043681 36835091 PMC9962028

[169] Zhong L Gan L Wang B Wu T Yao F Gong W. Hyperacute rejection-engineered oncolytic virus for interventional clinical trial in refractory cancer patients. Cell. (2025) 188:1119–1136.e1123. doi: 10.1016/j.cell.2024.12.010 39826543

[170] Osali A Zhiani M Ghaebi M Meymanat M Esmaeilzadeh A. Multidirectional strategies for targeted delivery of oncolytic viruses by tumor infiltrating immune cells. Pharmacol Res. (2020) 161:105094. doi: 10.1016/j.phrs.2020.105094 32795509

[171] Shahini L Hoxha M Marku F Morina B Cena V Kabashi K. Role of cytoblock on pleural effusion for diagnosis of Malignant disease. Diagn Cytopathol. (2023) 51:684–8. doi: 10.1002/dc.25201 37547992

[172] Nemet M Vasilić M Ergelašev S Kuhajda I Ergelašev I. Intrapleural fibrinolytic therapy with alteplase for the management of multiloculated Malignant pleural effusion: A case series. Cureus. (2022) 14:e27549. doi: 10.7759/cureus.27549 36059301 PMC9428410

[173] Shen CF Burney E Gilbert R Elahi SM Parato K Loignon M. Development, optimization, and scale-up of suspension Vero cell culture process for high titer production of oncolytic herpes simplex virus-1. Biotechnol J. (2024) 19:e2300244. doi: 10.1002/biot.202300244 37767876

[174] Moroz VD Gasanov NB Egorov AD Malogolovkin AS Nagornykh MO Subcheva EN. A method for the production of recombinant VSVs with confirmation of biological activity. Acta Naturae. (2024) 16:59–66. doi: 10.32607/actanaturae.27314 38698956 PMC11062106

[175] Torres-Ortega PV Smerdou C Ansorena E Ballesteros-Briones MC Martisova E Garbayo E. Optimization of a GDNF production method based on Semliki Forest virus vector. Eur J Pharm Sci. (2021) 159:105726. doi: 10.1016/j.ejps.2021.105726 33482318

[176] Göbel S Jaén KE Fernandes RP Reiter M Altomonte J Reichl U. Characterization of a quail suspension cell line for production of a fusogenic oncolytic virus. Biotechnol Bioeng. (2023) 120:3335–46. doi: 10.1002/bit.28530 37584190

[177] Swartz AR Shieh Y Gulasarian A Curtis E Hofmann CF Baker JB. Glutathione affinity chromatography for the scalable purification of an oncolytic virus immunotherapy from microcarrier cell culture. Front Bioeng Biotechnol. (2023) 11:1193454. doi: 10.3389/fbioe.2023.1193454 37397964 PMC10310922

[178] Gerstweiler L Billakanti J Bi J Middelberg A. Comparative evaluation of integrated purification pathways for bacterial modular polyomavirus major capsid protein VP1 to produce virus-like particles using high throughput process technologies. J Chromatogr A. (2021) 1639:461924. doi: 10.1016/j.chroma.2021.461924 33545579 PMC7825977

[179] Swartz AR Shieh Y Gulasarian A Olson JW Rustandi RR. Binding of Coxsackievirus A21 procapsids to immobilized glutathione depends on cell culture conditions during infection. Virology. (2022) 573:167–75. doi: 10.1016/j.virol.2022.06.013 35834888

[180] Gautam S Xin D Garcia AP Spiesschaert B. Single-step rapid chromatographic purification and characterization of clinical stage oncolytic VSV-GP. Front Bioeng Biotechnol. (2022) 10:992069. doi: 10.3389/fbioe.2022.992069 36394051 PMC9649487

[181] Mendes JP Silva RJS Berg M Mathiasson L Peixoto C Alves PM. Oncolytic virus purification with periodic counter-current chromatography. Biotechnol Bioeng. (2021) 118:3522–32. doi: 10.1002/bit.27779 33818758

[182] Turco F Wegelius A Lind O Norrman N Magnusson AC Sund-Lundström C. Combined clarification and affinity capture using magnetic resin enables efficient separation of rAAV5 from cell lysate. Mol Ther Methods Clin Dev. (2023) 30:394–402. doi: 10.1016/j.omtm.2023.07.010 37637382 PMC10457685

[183] Lorenzo E Miranda L Gòdia F Cervera L. Downstream process design for Gag HIV-1 based virus-like particles. Biotechnol Bioeng. (2023) 120:2672–84. doi: 10.1002/bit.28419 37148527

[184] Wang X Shen Y Wan X Hu X Cai WQ Wu Z. Oncolytic virotherapy evolved into the fourth generation as tumor immunotherapy. J Transl Med. (2023) 21(1):500. doi: 10.1186/s12967-023-04360-8 37491263 PMC10369732

[185] Hong Y Cheng K Qu H Wang Y Wang Y Fan G. Safety of talimogene laherparepvec: a real-world retrospective pharmacovigilance study based on FDA Adverse Event Reporting System (FAERS). J Pharm Health Care Sci. (2024) 10:79. doi: 10.1186/s40780-024-00388-0 39696696 PMC11654148

[186] Wang Y Zhou X Wu Z Hu H Jin J Hu Y. Preclinical safety evaluation of oncolytic herpes simplex virus type 2. Hum Gene Ther. (2019) 30:651–60. doi: 10.1089/hum.2018.170 30499341

[187] Wang Y Wang R Hu H Jin J Cai L Zhang S. Preclinical safety assessment of an oncolytic herpes simplex virus type 2 expressed PD-L1/CD3 bispecific antibody. Int Immunopharmacol. (2023) 124:110975. doi: 10.1016/j.intimp.2023.110975 37757634

[188] Dong J Kong L Wang S Xia M Zhang Y Wu J. Oncolytic adenovirus encoding apolipoprotein A1 suppresses metastasis of triple-negative breast cancer in mice. J Exp Clin Cancer Res. (2024) 43:102. doi: 10.1186/s13046-024-03011-0 38566092 PMC10988920

[189] Futami M Sato K Miyazaki K Suzuki K Nakamura T Tojo A. Efficacy and safety of doubly-Regulated vaccinia virus in a mouse xenograft model of multiple myeloma. Mol Ther Oncolytics. (2017) 6:57–68. doi: 10.1016/j.omto.2017.07.001 28808676 PMC5545772

[190] Zhang H Li K Lin Y Xing F Xiao X Cai J. Targeting VCP enhances anticancer activity of oncolytic virus M1 in hepatocellular carcinoma. Sci Transl Med. (2017) 9:eaam7996. doi: 10.1126/scitranslmed.aam7996 28835517

[191] Hoang HD Said A Vaidya N Gilchrist VH Malone K Kabilan U. Adaptation of transgene mRNA translation boosts the anticancer efficacy of oncolytic HSV1. J Immunother Cancer. (2023) 11:e006408. doi: 10.1136/jitc-2022-006408 36958764 PMC10040010

[192] Jirovec E Quixabeira DCA Clubb JHA Pakola SA Kudling T Arias V. Single intravenous administration of oncolytic adenovirus TILT-123 results in systemic tumor transduction and immune response in patients with advanced solid tumors. J Exp Clin Cancer Res. (2024) 43:297. doi: 10.1186/s13046-024-03219-0 39506856 PMC11539705

[193] Shi Z Liu B Huang C Xie W Cen Y Chen L. An oncolytic vaccinia virus armed with anti-human-PD-1 antibody and anti-human-4-1BB antibody double genes for cancer-targeted therapy. Biochem Biophys Res Commun. (2021) 559:176–82. doi: 10.1016/j.bbrc.2021.04.078 33945995

[194] Kasala D Lee SH Hong J Oh E Yoon AR Yun CO. Bioreducible polymer-mediated delivery of oncolytic adenovirus can attenuate antiviral immune response and concurrently enhance the induction of antitumor immune response to effectively prevent metastasis. Biomater Sci. (2022) 10:4293–308. doi: 10.1039/d2bm00200k 35766864

